# Cultured Bacteria Provide Insight into the Functional Potential of the Coral-Associated Microbiome

**DOI:** 10.1128/msystems.00327-22

**Published:** 2022-06-13

**Authors:** Jie Li, Yiyang Zou, Jian Yang, Qiqi Li, David G. Bourne, Michael Sweet, Cong Liu, Anjie Guo, Si Zhang

**Affiliations:** a CAS Key Laboratory of Tropical Marine Bio-resources and Ecology, South China Sea Institute of Oceanology, Chinese Academy of Sciences, Guangzhou, Guangdong, China; b Key Laboratory of Tropical Marine Biotechnology of Hainan Province, Sanya Institute of Oceanology, South China Sea Institute of Oceanology, Chinese Academy of Sciences, Guangzhou, Guangdong, China; c Sanya National Marine Ecosystem Research Station, South China Sea Institute of Oceanology, Chinese Academy of Sciences, Guangzhou, Guangdong, China; d College of Science and Engineering, James Cook University, Townsville, Queensland, Australia; e Australian Institute of Marine Science, Townsville, Queensland, Australia; f Aquatic Research Facility, Environmental Sustainability Research Centre, University of Derby, Derby, United Kingdom; Max Planck Institute for Marine Microbiology

**Keywords:** coral-associated bacteria, culture collection, novel taxon, genome sequencing, metabolic potential, coral-bacterium symbiosis

## Abstract

Improving the availability of representative isolates from the coral microbiome is essential for investigating symbiotic mechanisms and applying beneficial microorganisms to improve coral health. However, few studies have explored the diversity of bacteria which can be isolated from a single species. Here, we isolated a total of 395 bacterial strains affiliated with 49 families across nine classes from the coral Pocillopora damicornis. Identification results showed that most of the strains represent potential novel bacterial species or genera. We also sequenced and assembled the genomes of 118 of these isolates, and then the putative functions of these isolates were identified based on genetic signatures derived from the genomes and this information was combined with isolate-specific phenotypic data. Genomic information derived from the isolates identified putative functions including nitrification and denitrification, dimethylsulfoniopropionate transformation, and supply of fixed carbon, amino acids, and B vitamins which may support their eukaryotic partners. Furthermore, the isolates contained genes associated with chemotaxis, biofilm formation, quorum sensing, membrane transport, signal transduction, and eukaryote-like repeat-containing and cell-cell attachment proteins, all of which potentially help the bacterium establish association with the coral host. Our work expands on the existing culture collection of coral-associated bacteria and provides important information on the metabolic potential of these isolates which can be used to refine understanding of the role of bacteria in coral health and are now available to be applied to novel strategies aimed at improving coral resilience through microbiome manipulation.

**IMPORTANCE** Microbes underpin the health of corals which are the building blocks of diverse and productive reef ecosystems. Studying the culturable fraction of coral-associated bacteria has received less attention in recent times than using culture-independent molecular methods. However, the genomic and phenotypic characterization of isolated strains allows assessment of their functional role in underpinning coral health and identification of beneficial microbes for microbiome manipulation. Here, we isolated 395 bacterial strains from tissues of *Pocillopora damicornis* with many representing potentially novel taxa and therefore providing a significant contribution to coral microbiology through greatly enlarging the existing cultured coral-associated bacterial bank. Through analysis of the genomes obtained in this study for the coral-associated bacteria and coral host, we elucidate putative metabolic linkages and symbiotic establishment. The results of this study will help to elucidate the role of specific isolates in coral health and provide beneficial microbes for efforts aimed at improving coral health.

## INTRODUCTION

Genetic and genomic approaches have provided an in-depth understanding of the coral-associated microbiome. However, despite the diversity of the coral microbiome being well described (1), the functions these communities play regarding host fitness and health, or indeed the role of single members, remain less well understood (2–4). To this end, high-quality assembled reference genomes derived from metagenomic analyses are now desirable, thus enabling a better understanding of the integrated metabolic links of the coral holobiont (5). However, the often-close symbiotic relationships bacteria have with their coral hosts make it difficult to eliminate contaminating coral DNA for subsequent analyses (6). Studies now, however, are starting to overcome these issues, with recent work outlining 102 metagenome-assembled or single cell-sequenced bacterial genomes, along with 103 genomes derived from the cultured fraction of coral-associated bacteria (summarized in Data Set S2 at http://data.scsio.ac.cn/metaData-detail/1514896851117494272 and http://isolates.reefgenomics.org/download2/).

Although culture-independent molecular methods are useful in describing the diversity of any given host, the isolation and characterization of cultured fractions are still vital to elucidate strain characteristics. A previous report (7) highlighted the wealth of information which can be mined from such collections, identifying over 3,000 coral-associated bacteria held in laboratories around the world, 1,045 of which had full-length 16S rRNA gene sequences available. The study also increased the number of genomes available for these coral associates to 74 at the time of publication (7). However, there remains little understanding of culturable bacteria from a given coral species at a single time point (7). In this study, we investigate in detail the cultural fraction of Pocillopora damicornis, describing novel bacterial taxa associated with this coral species. Furthermore, through sequencing and analysis of genomes of these isolates and the coral host, we provide insight into the putative function these isolates offer to their host.

## RESULTS

### Cultured coral-associated bacteria reveal novel diversity.

**FIG 1:**
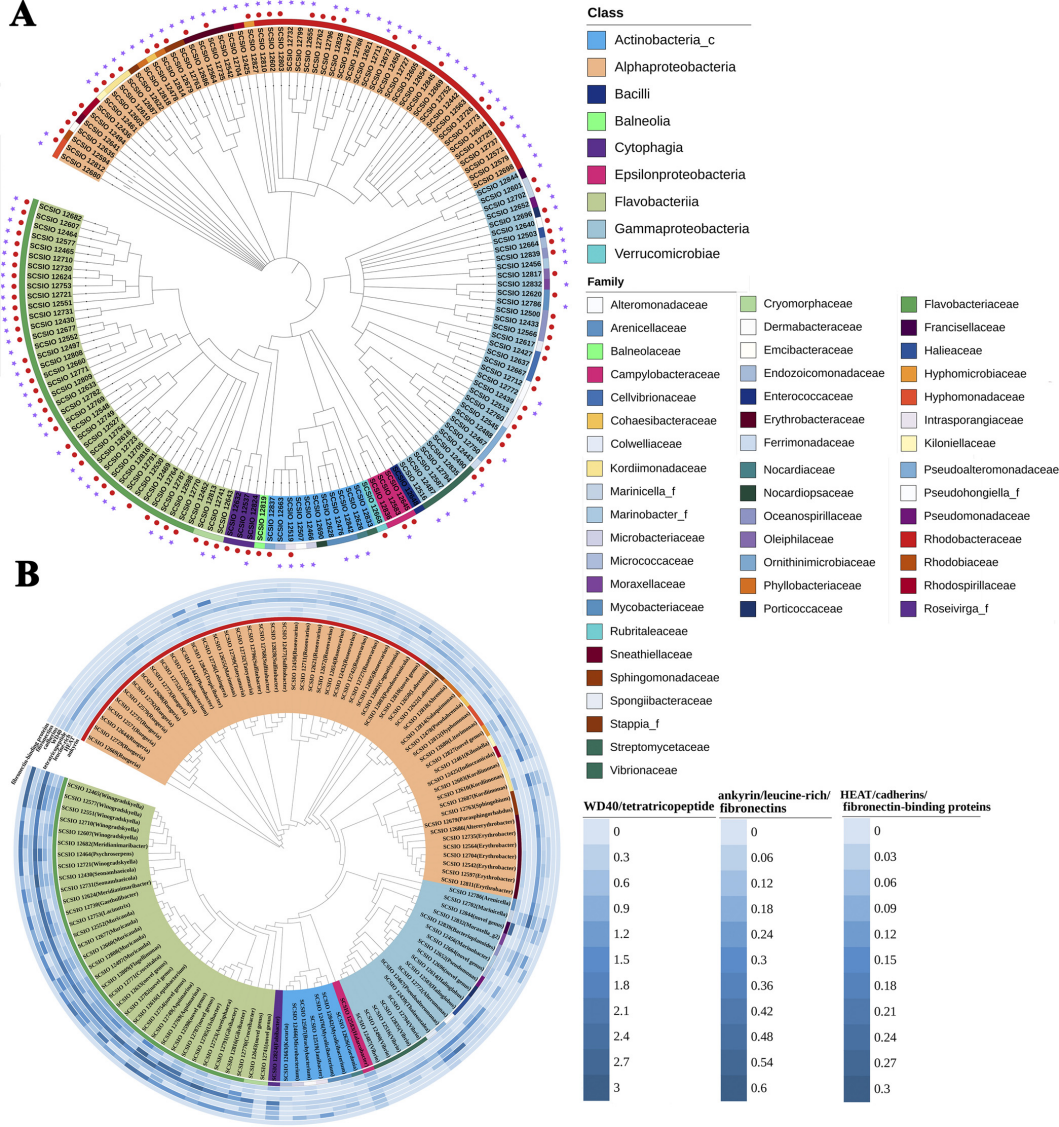
(A) Phylogenetic tree of representatives of 395 isolated coral-associated bacteria based on 16S rRNA gene sequences. The 395 strains are classified into 154 species (16S rRNA gene similarity, ≥98.7%) from 49 families and 9 classes. The strain number is color coded according to classes. The external ring is color coded according to families. The strains with sequenced genomes are marked with purple star symbols, and the 98 new species are indicated with red dots. (B) Phylogenomic tree of all coral-associated bacterial genomes within the collection and the distribution of eukaryote-like repeat proteins including ankyrin, leucine-rich, tetratricopeptide, HEAT, and WD40 as well as cadherins, fibronectins, and fibronectin-binding proteins. Values represent the percentage of coding genes in each class per genome. The inner ring is color coded according to families. The strain number is color coded according to classes. The genus name is included in parentheses, and “novel genus” shows the strain represents a candidate novel genus.

Using the dilution plating procedure on marine agar (MA), we obtained a total of 395 isolates from tissue of the coral *P. damicornis*. These isolates were classified into 49 families based on nearly full-length 16S rRNA gene sequences, across nine classes. These included *Actinobacteria* (14 isolates), *Cytophagia* (10 isolates), *Flavobacteriia* (87 isolates), *Bacilli* (1 isolate), *Alphaproteobacteria* (193 isolates), *Epsilonproteobacteria* (5 isolates), *Gammaproteobacteria* (80 isolates), *Balneolia* (1 isolate), and *Verrucomicrobiae* (4 isolates). The most abundant taxa cultured belonged to *Flavobacteriaceae* (84 isolates), *Rhodobacteraceae* (145 isolates), and *Gammaproteobacteria* (80 isolates) (Fig. 1A; see also Table S6 at http://data.scsio.ac.cn/metaData-detail/1514896851117494272 and http://isolates.reefgenomics.org/download2/). Local BLAST analysis of the detected operational taxonomic units (OTUs) from *P. damicornis* tissue collected on the same reef (8) against these bacterial cultures revealed 39 hits at the species level (>98.7% identity), which account for ~8% of the coral bacterial community (i.e., 39 out of 490 OTUs).

Using the stringent 98.7% 16S rRNA gene sequence similarity, we identified 154 distinct bacterial species (Fig. 1A; see also Table S6 at http://data.scsio.ac.cn/metaData-detail/1514896851117494272 and http://isolates.reefgenomics.org/download2/). Only 56 of these matched previously described species, suggesting the remaining isolates might represent novel taxa (Fig. 1A; see also Table S6). Among these, 80 are potential novel species (when matched against the EzBioCloud database [9]), and 18 represent 17 novel genera (there were 2 species affiliated with the same genus)—i.e., their 16S rRNA gene sequence similarity was <94.5% (see Table S6).

### Genome characteristics and functional gene distribution in coral-associated bacteria.

A total of 118 genomes were assembled from the cultured bacteria, and these accounted for 111 out of the 154 distinct taxa identified by 16S rRNA gene analysis alone (Fig. 1A and B; see also Table S6 at http://data.scsio.ac.cn/metaData-detail/1514896851117494272 and http://isolates.reefgenomics.org/download2/). While nine of these 118 genomes are complete, the remaining draft genomes have a completeness of ≥97.1% (see Data Set S3 at http://data.scsio.ac.cn/metaData-detail/1514896851117494272 and http://isolates.reefgenomics.org/download2/). Moreover, 13 isolates possess 2 to 5 copies of the 16S rRNA gene, and the divergences were below 0.002. The results of digital DNA-DNA hybridization (dDDH) and average nucleotide identity (ANI) analyses based on the genomes of 66 candidate novel species further support separating them from any previously described species (see Table S6).

**FIG 2:**
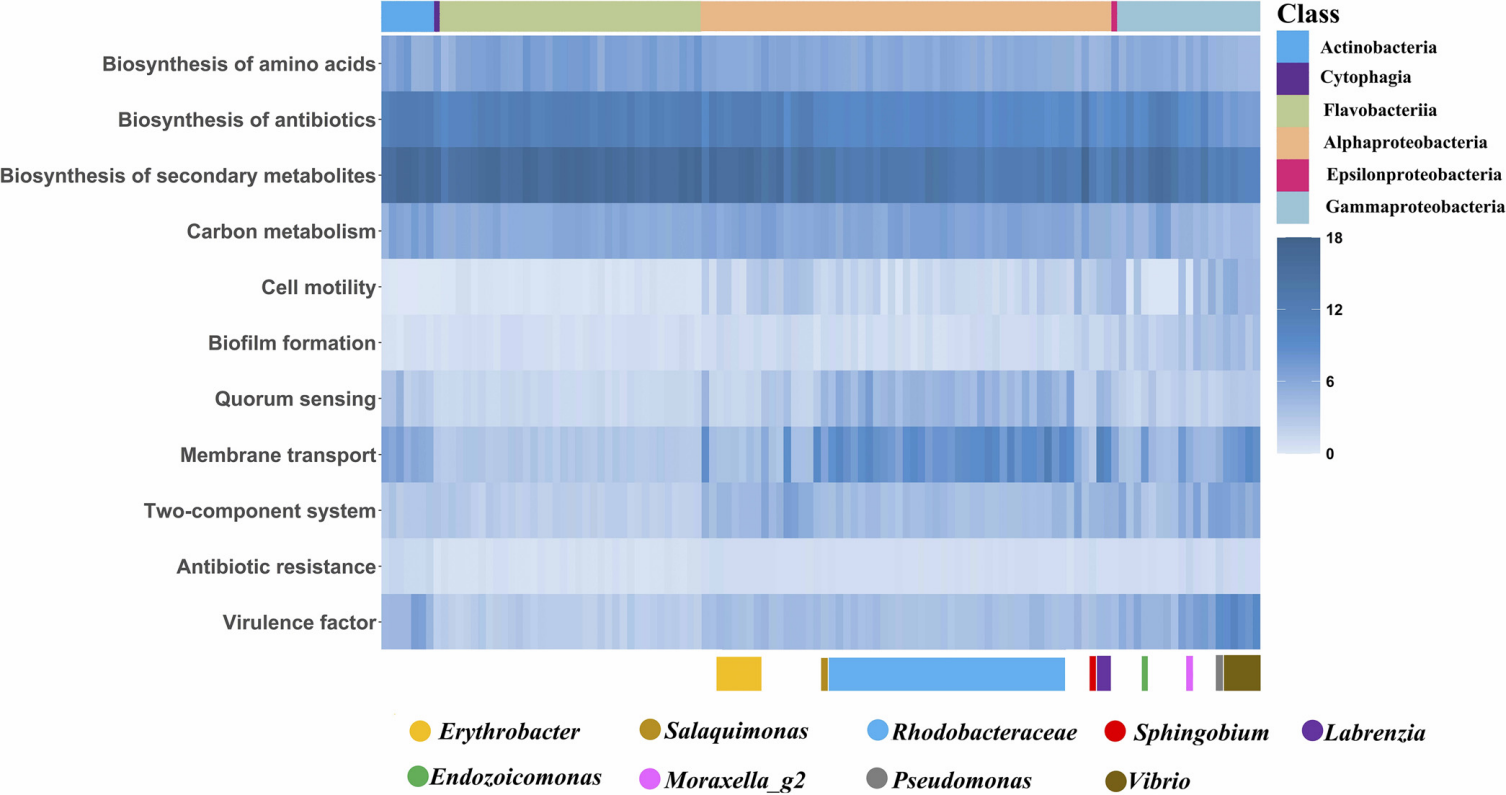
Functional landscape of coral-associated bacteria. Heat map representing the gene abundance of the listed functions in the 118 genomes of coral-associated bacteria.

Putative metabolic functions of these bacteria, assessed using Kyoto Encyclopedia of Genes and Genomes (KEGG) level 2 assignments, indicated that carbohydrate and amino acid metabolic pathways were the most abundant in all isolates (see Fig. S1 in the supplemental material). Further, energy metabolism and metabolism of cofactors and vitamins were also abundant (Fig. 2; Fig. S1; see also Data Set S4 at http://data.scsio.ac.cn/metaData-detail/1514896851117494272 and http://isolates.reefgenomics.org/download2/). A focus on the pathways showing class- or genus-specific distribution (Fig. 2; see also Data Set S4) demonstrated that biosynthesis of amino acids, antibiotics, and secondary metabolites and carbon metabolism were ubiquitous and abundant in all isolates. Cell motility (chemotaxis, ko02030; flagellar assembly, ko02040) was conserved mainly in the phylum *Proteobacteria*. Genes involved in biofilm formation were mainly found in the *Gammaproteobacteria*. *Alphaproteobacteria* showed a relatively high abundance of genes involved in quorum sensing, especially in the family *Rhodobacteraceae* and the genera *Labrenzia* and *Salaquimonas* (Fig. 2). Genes involved in membrane transport (mainly ABC transporters) were abundant in most genomes of the class *Alphaproteobacteria*, the genus *Vibrio*, and strain SCSIO 12664, which represents a novel genus in the family *Endozoicomonadaceae* (Fig. 2). The signal transduction system (two-component system, ko02020) was abundant in *Proteobacteria* and is potentially involved in sensing and responding to altered environmental conditions.

10.1128/msystems.00327-22.1 FIG S1 Distribution of functional pathways in different bacterial classes of the coral-associated microbiota. The *x* axis indicates the different bacterial classes, and the *y* axis indicates the functional pathways at KEGG levels 1 and 2. Download FIG S1, TIF file, 2.7 MB. Copyright © 2022 Li et al. 2022 Li et al. https://creativecommons.org/licenses/by/4.0/ This content is distributed under the terms of the Creative Commons Attribution 4.0 International license.

Antibiotic resistance was abundant in the *Actinobacteria* phylum and in most isolates of the *Proteobacteria* phylum, especially in *Erythrobacter*, *Sphingobium* (*Alphaproteobacteria*), *Moraxella*_g2, Pseudomonas, and *Vibrio* (*Gammaproteobacteria*) (Fig. 2; see also Data Set S4 at http://data.scsio.ac.cn/metaData-detail/1514896851117494272 and http://isolates.reefgenomics.org/download2/). A type III secretion system, which is known to be essential for the interaction of Gram-negative bacteria with eukaryotic hosts (10), was identified in several of our isolates affiliated with *Pseudoalteromonas*, *Vibrio*, and *Endozoicomonadaceae*. Putative virulence factors were abundant in the class *Gammaproteobacteria*, especially in the genera Pseudomonas and *Vibrio* (Fig. 2; see also Data Set S4). Additionally, the protein family profiles of our isolated vibrios were distinct from previously identified nonpathogenic *Vibrio* (permutational multivariate analysis of variance [PERMANOVA], *R*^2^ = 0.221, F = 2.551, *P* < 0.01), while they had no significant difference from pathogenic *Vibrio* (PERMANOVA, *R*^2^ = 0.07, F = 0.608, *P* > 0.1) (Fig. S2).

10.1128/msystems.00327-22.2 FIG S2 Principal-coordinate analysis (PCoA) of the protein family profiles of *Vibrio* strains using the Bray-Curtis distance matrix calculated from the Hellinger-transformed abundance data according to the method provided by Sweet et al. (7). Pink dots represent nonpathogenic strains; green dots represent pathogenic strains; blue dots represent strains cultured in this study. Download FIG S2, TIF file, 2.3 MB. Copyright © 2022 Li et al. 2022 Li et al. https://creativecommons.org/licenses/by/4.0/ This content is distributed under the terms of the Creative Commons Attribution 4.0 International license.

### ELR-containing and cell-cell attachment proteins.

Eukaryote-like repeat (ELR)-containing proteins from the ankyrin (ARP), WD40, leucine-rich, tetratricopeptide, and HEAT repeat families are thought to modulate the host’s intracellular processes and have a role in stable symbiosis (5, 11–14). In this study, we found them to be more prevalent in members of the *Flavobacteriaceae*, while ELR abundances were generally lower in *Actinobacteria* and *Alphaproteobacteria*. In comparison to the other four types of ELR, the tetratricopeptide was more abundant in our isolates (Fig. 1B). Two isolates in particular, *Aquimarina* sp. (SCSIO 12749) and *Francisellaceae* (SCSIO 12844), devoted 0.3% and 0.5%, respectively, of their coding genes to ARPs (Fig. 1B). The WD40-repeat-containing proteins were most abundant in a *Fabibacter* (SCSIO 12824) with 51 copies while other isolates showed between 2 and 38 copies in comparison. Leucine-rich repeats represented less than 0.55% in our isolates. Interestingly, both leucine-rich and HEAT repeats were absent in approximately half of our isolates, specifically those belonging to *Alphaproteobacteria*, *Gammaproteobacteria*, and *Actinobacteria* (Fig. 1B).

Cadherins, a superfamily of transmembrane glycoproteins, mediate Ca^2+^-dependent cell-cell adhesion throughout the animal kingdom and have been found to mediate bacterial cell-cell contact in the ocean (15). Here, we identified genes containing cadherin domains in more than half of our isolates across all lineages (Fig. 1B). In the coral host genome, we also identified fibronectin type II- and III-encoding genes (Table S1). Fibronectin-binding proteins (FnBPs) are considered essential for attachment of host-associated bacteria to their ecological niches by binding to host fibronectin (16). Surprisingly, only three of our isolates (*Thalassotalea* sp. strain SCSIO 12439, *Halarcobacter* sp. strain SCSIO 12583, and *Ruegeria* sp. strain SCSIO 12644) were found to possess genes encoding FnBPs specifically. That said, genes encoding fibronectin type III were found distributed in more than half of our isolates, including all *Bacteroidetes* and several *Proteobacteria* and *Actinobacteria* members (Fig. 1B).

10.1128/msystems.00327-22.5 TABLE S1 Putative fibronectin-related genes in the coral genome. Download Table S1, XLSX file, 0.03 MB. Copyright © 2022 Li et al. 2022 Li et al. https://creativecommons.org/licenses/by/4.0/ This content is distributed under the terms of the Creative Commons Attribution 4.0 International license.

### Carbon, nitrogen, and sulfur cycling.

**FIG 3:**
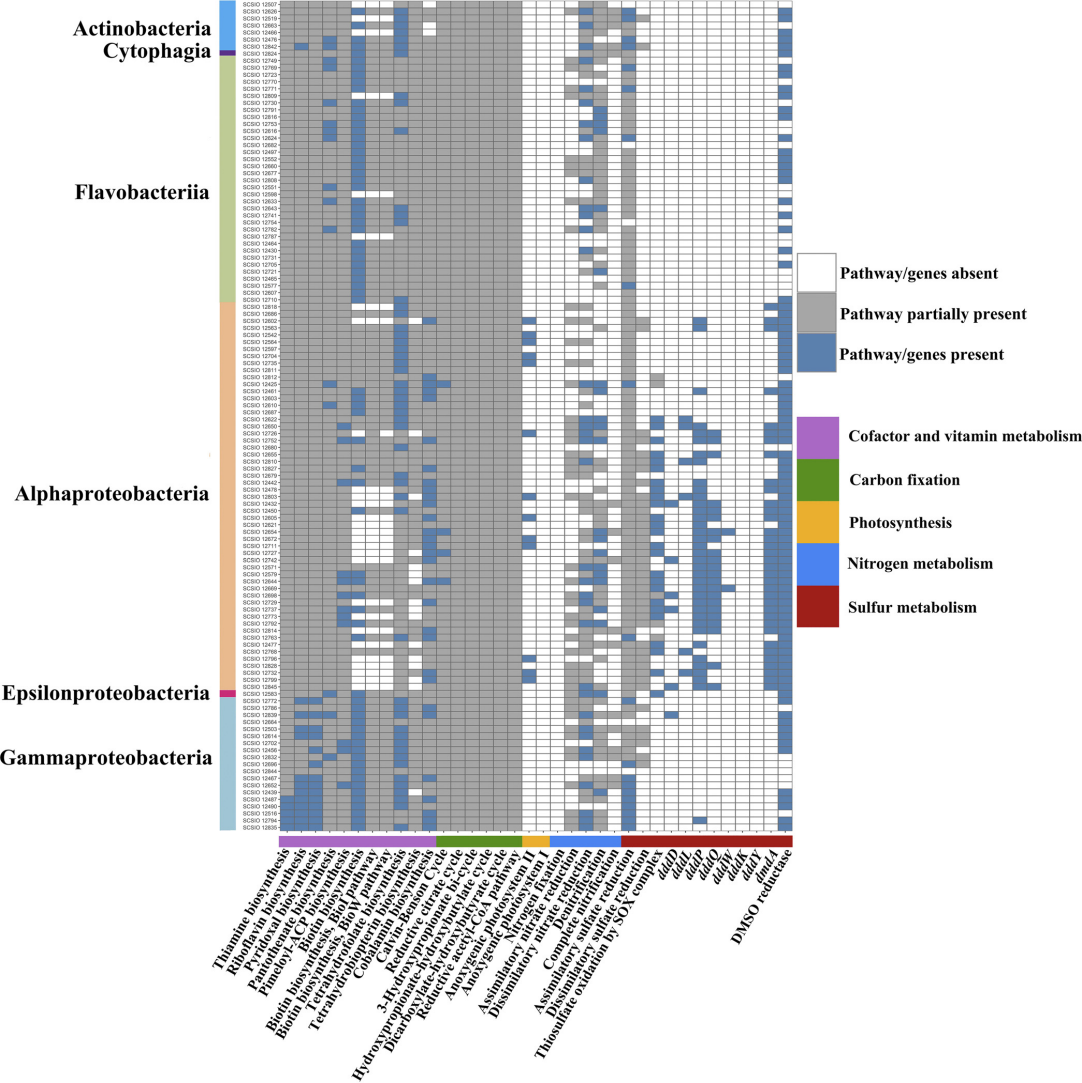
Color-coded table indicating major functional genes and their abundance in coral-associated bacterial genomes. The *x* axis indicates the genes likely involved in energy or vitamin metabolism, and the *y* axis indicates the strain designations. Blue indicates the complete pathway identified in carbon, sulfur, and nitrogen nutrient cycles and vitamin biosynthesis and the genes present in DMSP cleavage (*dddD*, *dddL*, *dddP*, *dddQ*, *dddW*, *dddK*, *dddY*, and *dmdA*) and DMSO reduction; gray indicates the partial pathway identified in the carbon, sulfur, and nitrogen nutrient cycles and vitamin biosynthesis; white indicates an absence of relevant genes or pathways.

A total of 13 *Alphaproteobacteria* isolates from the genera *Cognatiyoonia*, *Erythrobacter*, *Leisingera*, *Pseudooceanicola*, *Roseovarius*, *Sulfitobacter*, and *Tateyamaria* possess both the L (*pufL*) and M (*pufM*) subunits of the photosynthetic reaction center, constituting the anoxygenic photosystem II (Fig. 3; see also Table S7 at http://data.scsio.ac.cn/metaData-detail/1514896851117494272 and http://isolates.reefgenomics.org/download2/).

A range of carbon fixation pathways were identified in the present study, including the Calvin-Benson cycle, Arnon-Buchanan cycle, 3-hydroxypropionate bi-cycle, hydroxypropionate-hydroxybutylate cycle, dicarboxylate-hydroxybutyrate cycle, and Wood-Ljungdahl pathway (Fig. 3; see also Table S7 at http://data.scsio.ac.cn/metaData-detail/1514896851117494272 and http://isolates.reefgenomics.org/download2/). The complete sets of genes encoding the Calvin-Benson cycle were found in strains SCSIO 12425 (*Indioceanicola*), SCSIO 12644 (*Ruegeria*), and SCSIO 12654 and SCSIO 12727 (*Roseovarius*) (Fig. 3; see also Table S7).

Diazotrophs have been suggested to supply nitrogen to the host and the algal symbionts (17); however, genes encoding nitrogenases were not found in this study (Fig. 3). Additionally, there was no evidence of ammonia oxidation-related genes in our isolates, though nitrogen can be acquired from the surrounding seawater (5, 18) or derived from the coral host directly (19). That said, nitrite can be oxidized to nitrate, and this appears possible from 16 of our isolates from the genera *Gordonia*, *Janibacter*, *Kocuria*, *Mycolicibacterium*, *Fabibacter*, *Mesorhizobium*, *Roseovarius*, *Sulfitobacter*, *Bacterioplanoides*, *Halioglobus*, *Marinobacter*, *Moraxella*_g2, *Pseudoalteromonas*, and Pseudomonas, all of which possess the alpha (*nxrA*) and beta (*nxrB*) subunit of the nitrite oxidoreductase NXR. Further, we found the complete sets of genes encoding the dissimilatory nitrate reduction pathway in 35 isolates (Fig. 3; see also Table S7 at http://data.scsio.ac.cn/metaData-detail/1514896851117494272 and http://isolates.reefgenomics.org/download2/). and the complete denitrification pathway was identified in 19 (Fig. 3; see also Table S7).

Specifically, strains SCSIO 12603, SCSIO 12610, and SCSIO 12664 showed nitrate reduction activity (see Data Set S1, the description of the novel taxon), although the corresponding KEGG modules were incomplete (Fig. 3; see also Table S7 at http://data.scsio.ac.cn/metaData-detail/1514896851117494272 and http://isolates.reefgenomics.org/download2/). In contrast, the complete sets of genes encoding the dissimilatory nitrate reduction and/or denitrification pathways were observed in three other strains, SCSIO 12643, SCSIO 12741, and SCSIO 12839 (Fig. 3; see also Table S7), while they did not show nitrate reduction activity in the biochemical assays (see Data Set S1, the description of the novel taxon).

10.1128/msystems.00327-22.10 DATA SET S1 Description of novel taxa. Download Data Set S1, PDF file, 2.7 MB. Copyright © 2022 Li et al. 2022 Li et al. https://creativecommons.org/licenses/by/4.0/ This content is distributed under the terms of the Creative Commons Attribution 4.0 International license.

Dimethylsulfoniopropionate (DMSP) lyases (*dddD*, *dddL*, *dddP*, *dddQ*, and *dddW*) and demethylases (*dmdA*) were found present in some of our isolates, mainly in the class *Alphaproteobacteria* and especially the family *Rhodobacteraceae*. Interestingly, *dddK* and *dddY* were not detected in any of our isolates (Fig. 3). Dimethyl sulfoxide (DMSO) reductase, in contrast, was more widely distributed across our isolates, present in 90 out of the 118 sequenced (Fig. 3).

Inorganic sulfur is also important for the coral holobiont, with the sulfur-based amino acid production central to sulfate metabolism (20). Complete sets of genes responsible for assimilatory sulfate reduction were identified in 20 of our isolates, half of which belonged to *Gammaproteobacteria* (Fig. 3; see also Table S7 at http://data.scsio.ac.cn/metaData-detail/1514896851117494272 and http://isolates.reefgenomics.org/download2/). No complete dissimilatory sulfate reduction pathway was observed in our isolates; however, the gene *sat*, which encodes sulfate adenylyltransferase involved in the dissimilatory sulfate reduction pathway, was present in all *Rhodobacteraceae* and several *Actinobacteria* and *Gammaproteobacteria* strains (Fig. 3; see also Table S7). Thiosulfate oxidation by the complete SOX complex was present in nearly half of the *Alphaproteobacteria* strains and in one (SCSIO 12583) belonging to the genus *Halarcobacter* (Fig. 3; see also Table S7).

### CAZy family and carbon metabolism.

**FIG 4:**
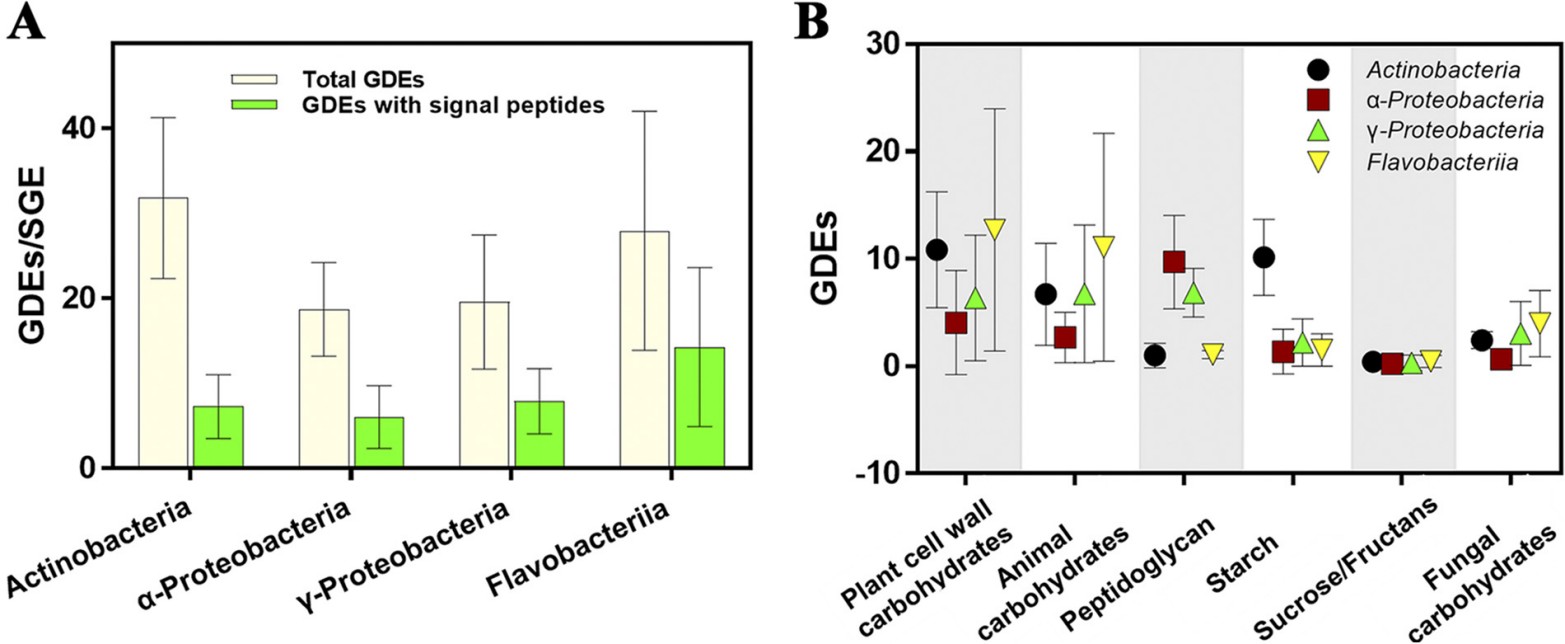
Carbohydrate utilization potential of coral-associated bacteria. (A) Frequency of glycoside-degrading enzyme sequences per sequenced genome equivalent. (B) Contribution of major potential degrader clades to the pool of sequences targeting specific carbohydrate substrates.

To evaluate the utilization potential for carbohydrates derived from coral-associated bacteria, we identified 3,756 potential glycoside-degrading enzymes (GDEs), including 434 auxiliary activities (AAs), 468 carbohydrate esterases (CEs), 2,696 glycoside hydrolases (GHs), and 107 polysaccharide lyases (PLs), across the 118 sequenced bacterial genomes. We found that the GDE content was variable across phyla with the average frequency of GDE sequences ranging from six (*Epsilonproteobacteria*) to 31.8 (*Actinobacteria*) per sequenced genome equivalent (SGE) (21) (i.e., 3 Mbp) (Fig. S3). The *Bacteroidetes* exhibited a similar overall frequency of GDEs (27.95) as the *Actinobacteria*; however, they held much higher content of extracellular carbohydrate-degrading enzymes (Fig. 4A). The lowest GDE frequency (with a total of six GDEs—five GHs and one CE) was detected in the genome of *Halarcobacter* sp. strain SCSIO 12583. Interestingly, *Endozoicomonadaceae* SCSIO 12664, a likely endosymbiont of coral (22, 23), also exhibited fewer GDEs than most of the obtained isolates, with two CEs and eight GHs detected.

10.1128/msystems.00327-22.3 FIG S3 Distribution of glycoside-degrading enzymes in coral-associated bacteria. The *x* axis indicates different classes of glycoside-degrading enzymes, and the *y* axis indicates the strain designations. The bar shows the frequency of glycoside-degrading enzyme sequences per sequenced genome equivalent (i.e., 3 Mbp, SGE). Download FIG S3, TIF file, 2.8 MB. Copyright © 2022 Li et al. 2022 Li et al. https://creativecommons.org/licenses/by/4.0/ This content is distributed under the terms of the Creative Commons Attribution 4.0 International license.

The most prevalent GDEs belonged to families acting on plant cell wall carbohydrates (GH3 and GH16), animal carbohydrates (PL6), and peptidoglycan (GH23, GH73, and GH103 and CE4) (Fig. 4B; Table S2). Besides those enriched functions, other GH families, known to degrade major components of dissolved organic matter in coral reefs, were frequently detected, including GH33 and GH42, GH29 and GH95, and GH51 and GH127 targeting sialic acids, fucoidan, and arabinosaccharides, respectively.

10.1128/msystems.00327-22.6 TABLE S2 Broad substrate categories of CAZy families. Download Table S2, DOCX file, 0.02 MB. Copyright © 2022 Li et al. 2022 Li et al. https://creativecommons.org/licenses/by/4.0/ This content is distributed under the terms of the Creative Commons Attribution 4.0 International license.

CAZyme fingerprinting of the nine potentially novel species evaluated via the API ZYM kit showed that five (SCSIO 12603, SCSIO 12610, SCSIO 12741, SCSIO 12839, and SCSIO 12844) did not exhibit hydrolytic activity against the tested glycoside substrates, and one (SCSIO 12827 of *Hyphomicrobiaceae*) displayed a broad spectrum of CAZyme activities (Table S3). Strain SCSIO 12664 showed activities of α-galactosidase, β-galactosidase, and *N*-acetyl-β-glucosaminidase (Table S3). Additionally, no β-glucuronidase or β-fucosidase activity was detected for all the examined isolates (Table S3). This suggests that nutrients like uronic acids or fucose-containing carbohydrates are unavailable to these bacteria. Genomic analysis of the nine novel species showed an average 12 detected GDEs, implying that they might share common metabolic features with relatively poor carbohydrate utilization potential. These isolates could, however, utilize most of the tested oligosaccharides, especially SCSIO 12696, which may well use all the oligosaccharides with distinct residues as carbon sources (Table S3). It is also possible that some of the GDEs are multifunctional, i.e., they may catalyze the transformation of a broad range of substrates.

10.1128/msystems.00327-22.7 TABLE S3 Partial hydrolase activities and carbon source utilization of nine novel species and the numbers of glycoside-degrading enzymes detected in their genomes. Download Table S3, DOCX file, 0.02 MB. Copyright © 2022 Li et al. 2022 Li et al. https://creativecommons.org/licenses/by/4.0/ This content is distributed under the terms of the Creative Commons Attribution 4.0 International license.

### Biosynthesis of amino acids and vitamins.

According to the genome analysis of the coral host, *P. damicornis* lacks the capability to biosynthesize eight amino acids including isoleucine, leucine, lysine, methionine, phenylalanine, tryptophan, tyrosine, and valine (Table S4). However, this should be interpreted with caution as the host genome assembly was incomplete (completeness score, 94.2%). Interestingly, we did find that many of our isolates potentially synthesize all 20 amino acids (Table S4; see also Table S7 at http://data.scsio.ac.cn/metaData-detail/1514896851117494272 and http://isolates.reefgenomics.org/download2/). Additionally, 22 of the *Alphaproteobacteria* strains may synthesize putrescine from arginine, and 10 isolates belonging to *Winogradskyella*, *Alphaproteobacteria*, and *Gammaproteobacteria* may be able to synthesize spermidine from arginine (see Table S7). Further, 12 isolates belonging to *Actinobacteria*, *Flavobacteriaceae*, *Alphaproteobacteria*, and *Gammaproteobacteria* could potentially synthesize γ-aminobutyric acid (GABA) from putrescine, and a *Labrenzia* sp. (SCSIO 12622) possesses the complete set of genes involved in the biosynthesis of GABA from arginine (see Table S7).

10.1128/msystems.00327-22.8 TABLE S4 Genome capabilities of *Pocillopora damicornis* and bacterial symbionts in the biosynthesis of amino acids and vitamins. Download Table S4, DOCX file, 0.02 MB. Copyright © 2022 Li et al. 2022 Li et al. https://creativecommons.org/licenses/by/4.0/ This content is distributed under the terms of the Creative Commons Attribution 4.0 International license.

The coral host genome also lacks vitamins associated with biosynthesis capability (including biotin, cobalamin, folate, pantothenate, riboflavin, thiamine, and pyridoxine) (Table S4). The complete sets of genes responsible for biosynthesis, however, can be found in many of our isolates. For example, thiamine, riboflavin, and pyridoxal were mainly distributed in *Gammaproteobacteria* strains (Fig. 3; see also Table S7 at http://data.scsio.ac.cn/metaData-detail/1514896851117494272 and http://isolates.reefgenomics.org/download2/). Seven *Rhodobacteraceae* and three *Gammaproteobacteria* strains may be able to synthesize biotin via the BioC-BioH pathway (Fig. 3; see also Table S7). Pantothenate and tetrahydrofolate biosynthesis pathways were distributed across isolates from various phyla, while the complete set of genes for cobalamin biosynthesis was possessed just by *Proteobacteria* (Fig. 3; see also Table S7). Finally, we assessed the yields of vitamins of strains SCSIO 12696 and SCSIO 12664 and confirmed that they were able to produce vitamins including folic acid, biotin, and riboflavin; SCSIO 12696 could also produce cyanocobalamin, thiamine, and pantothenic acid (Table S5). These results give strong support to the role these isolates play in the coral host as an important and alternative source of vitamins.

10.1128/msystems.00327-22.9 TABLE S5 Yields of vitamins in extracellular and intracellular analyses of strains SCSIO 12696 and SCSIO 12664. Download Table S5, DOCX file, 0.02 MB. Copyright © 2022 Li et al. 2022 Li et al. https://creativecommons.org/licenses/by/4.0/ This content is distributed under the terms of the Creative Commons Attribution 4.0 International license.

### Partial phenotypic and genomic properties of nine novel taxa.

Six novel genera and nine novel species were polyphasically characterized via phylogenetic analysis, morphological observation, phenotypic characterization using Biolog and API ZYM tests, chemotaxonomic analyses, and genome comparison. Most of these novel isolates showed esterase, lipase, and trypsin activities (Table S3). Partial phenotypic and genomic characteristics of these nine novel taxa are presented in Table 1, and further descriptions of them are provided in Data Set S1.

**TABLE 1:** Partial phenotypic and genomic characteristics of nine novel taxa^*a*^

Characteristic	SCSIO 12603	SCSIO 12610	SCSIO 12643	SCSIO 12664	SCSIO 12696	SCSIO 12741	SCSIO 12827	SCSIO 12839	SCSIO 12844
Closest match (BLAST search with EzBioCloud’s Identify service)	Kordiimonas lipolytica	Kordiimonas lipolytica	Vicingus serpentipes	Endozoicomonas montiporae	Porticoccus litoralis	Owenweeksia hongkongensis	Methyloligella solikamskensis	Bacterioplanoides pacificum	Fangia hongkongensis
Motility	+	+	+	−	+	−	+	+	−
Cell shape	Rod or slightly curved rod	Rod	Rod	Curved rod or irregular	Rod or oval	Rod	Curved or rod	Curved rod or spiral	Rod
Cell length (μm)	1.6–2.5	1.5–3.2	1.4–2.3	3.1–4.6	1.1–1.7	0.8–3.3	1.3–2.6	2.0–6.4	1.0–3.0
Cell width (μm)	0.5–0.6	0.6–0.8	0.5–0.7	0.5–0.9	0.4–0.7	0.4–0.7	0.4–0.6	0.4–0.6	0.4–0.6
Temp range for growth (°C) (optimum)	10–33 (25)	15–37 (25)	15–35 (30–35)	20–35 (25)	10–35 (30–35)	20–35 (30)	20–42 (30)	15–37 (25–30)	15–37 (30)
pH range for growth	5–8	6–7	6–8	4–8	5–7	6–8	5–7	6–8	5–7
NaCl tolerance (%, wt/vol)	3	3–5	3–5	3–3.7	3–7	3–3.7	3–7	3–7	0.5–5
DNA G+C content (%)	46.02	45.72	36.37	46.77, 41.65 (plasmid)	51.98	44.21	62.98	47.92	32.92

a Symbols: +, positive; −, negative.

## DISCUSSION

### Diverse and novel bacteria cultured from *P. damicornis*.

In this study, we isolated 395 bacterial strains from a single coral species, *P. damicornis*. There were 80 potential novel species and 17 potential novel genera in this collection, which greatly increases the currently available cultured bacteria derived from *P. damicornis* and corals in general. For example, in a recent effort to describe the cultured fraction of coral-associated bacteria 3,055 isolates were identified spread across 52 studies or held in laboratories in private collections (7). Only 15 of these (0.5%) were from the coral *P. damicornis*, and these were affiliated with seven genera (7). Further, that meta-analytical study identified 12 putatively novel genera (7). In this study, our isolates span 36 formally described genera and 17 potential novel genera (see Fig. S4 in the supplemental material). And our study focused on just one coral species, therefore further demonstrating the high species diversity of the coral microbiome (not including Symbiodiniaceae) and highlighting that cultivation approaches are underutilized in exploring the coral microbiome.

10.1128/msystems.00327-22.4 FIG S4 Cultured coral-associated bacterial genera. Bacterial genera in the light blue circle were newly obtained in this study, and those in the light pink circle were collated in the previous study (7). Genera in the overlapping circle were common to both studies. Download FIG S4, TIF file, 0.3 MB. Copyright © 2022 Li et al. 2022 Li et al. https://creativecommons.org/licenses/by/4.0/ This content is distributed under the terms of the Creative Commons Attribution 4.0 International license.

In addition to the increase in available cultured coral-associated bacterial strains and 16S rRNA gene data, we also added 118 genomes of coral-associated bacteria to data banks, representing 111 bacterial species. Our efforts effectively more than double the number of coral-associated bacterial genomes now available to researchers around the world for open-source use. That said, even with this significant contribution the number of genomes available for coral-associated bacteria remains limited, and there is an urgent need to increase this resource, which would facilitate a greater understanding of the putative mechanisms for establishment, maintenance, and function of bacteria associated with their coral hosts.

### ELR-containing and cell-cell attachment proteins involved in symbiosis maintenance.

Across the taxa of strains for which genome assembly was conducted, we observed that ELR-containing proteins were more prevalent in *Flavobacteriaceae* than in *Actinobacteria* and *Alphaproteobacteria*, implying potentially different degrees of association within the coral host. The two *Aquimarina* strains had the overall highest number of ELRs, which is somewhat similar to Aquimarina megaterium EL33, an octocoral associate (7). Four *Alteromonadales* strains had high numbers of WD40 and tetratricopeptide repeats, which again confirms previous findings for other coral-associated *Alteromonadales* (i.e., >250 tetratricopeptide and 29 to 142 WD40 repeats [7]). The same was found for vibrios in this and previous (7) studies, in that no or only a few ARPs were detected. In contrast to the findings of Sweet et al. (7) and a metagenomic binning study by Robbins et al. (5), we did not find high numbers of ankyrin and WD40 repeat sequence signatures in our *Endozoicomonadaceae* strain (SCSIO 12664). This was surprising as *Endozoicomonas* has been proposed to be a true symbiont in many coral species, though it likely also has a free-living life stage (23, 24). The lower abundance or complete absence of ELRs in our novel genus may therefore imply that SCSIO 12664 is only loosely related to the coral host and/or that the bacterium utilizes alternative mechanisms to maintain a stable association with the host. That said, although the ELRs are indeed found to be prevalent in corals and other marine invertebrate bacterial symbionts, the mechanism by which ELRs actually interact with host cells and the outcomes of these interactions still remain largely unknown.

Understanding symbiosis is one key goal, and analyzing both the genomes of the corals and their associated bacteria allows us to explore integrated links that may support a symbiotic lifestyle. For example, eukaryotic-like proteins are known to be used by some bacteria to manipulate host cellular processes (12–14), as well as mechanisms for attachment to the host tissue (14, 16). For *P. damicornis* we were able to detect fibronectin type II- and III-encoding genes in the host genome, while FnBPs were found in only three of the bacterial isolates. We noticed most of our isolates actually possessed fibronectin type III-encoding genes. Fibronectin III domains are the bacterial binding sites and mediate self-interaction in eukaryotes but have also been found in bacterial FnBPs and may interact with the self-binding region of fibronectin (16, 25). The fibronectin III domains may thus play an important role in bacterium-bacterium adhesion, and the isolates containing fibronectin III-encoding genes may also possess distinctive mechanisms allowing for the binding of host fibronectin. Further investigation is therefore warranted in this regard and would ascertain the functions of these eukaryote-like and binding proteins, ultimately unlocking the key mechanisms involved in coral-bacterial symbiont interactions.

### Genomic and phenotypic support for the contribution to carbon and nitrogen cycling.

Besides acquiring carbon through zooplankton predation (heterotrophic feeding) and photosynthetic fixation by the endosymbiotic algae Symbiodiniaceae, several essential genes for carbon fixation pathways have been identified in the metagenome-assembled genomes of coral-associated bacteria and archaea (5). Here, we have also identified six carbon fixation pathways, and four isolates (SCSIO 12425, SCSIO 12644, SCSIO 12654, and SCSIO 12727) in particular were found to possess the complete sets of genes encoding the Calvin-Benson cycle. Our results imply the presence of bacterial carbon fixers in the holobiont (in addition to the Symbiodiniaceae). Of specific interest are the aerobic anoxygenic phototrophic bacteria (AAPB) observed in this study, which possess both *pufL* and *pufM* genes. To our knowledge, this is the first study reporting the potential presence of AAPB associated with coral based on the presence of *pufLM* genes, though expression of relevant gene pathways is still required to confirm whether this finding has any functional relevance to the coral holobiont. We hypothesize that these bacteria likely utilize light as an additional source of energy for metabolism (26), much the same way as the symbiotic algae, and therefore indirectly reduce the consumption of organic carbon. Additionally, light appears to stimulate and promote the cell activity of AAPB, thus promoting the carbon cycle process, which will have a knock-on effect for the host and other members of the coral microbiome (27).

It should be noted that although we did not isolate any diazotrophic microbes from *P. damicornis* tissues, this was likely due to the methodology utilized and does not imply they are not present. Similarly, our culture approaches would not retrieve the archaeal lineages (*Thaumarchaeota*), which have been implicated in metabolism of urea/ammonia to nitrate in corals (5). That said, we were able to identify several nitrifying and denitrifying bacteria which are likely contributing to the nitrogen cycling process within the coral holobiont. While the function of coral-associated bacteria was largely inferred based on our genomic data, we also conducted biochemical assays on some of the novel isolates, and three (SCSIO 12603, SCSIO 12610, and SCSIO 12664) revealed nitrate-reducing capabilities. Interestingly, there were some inconsistencies between the phenotypic and genomic evidence for some of these isolates, which highlights the necessity of functional verification when describing the roles of key coral-associated bacteria.

### CAZy family and carbon metabolism provide cues for microniche adaptation.

Differences in the chemical composition of labile organic matter exuded by coral holobionts influence the community and growth of associated bacteria, which can subsequently differentially enrich bacterial lineages (28). Microbial GDEs from GH23, GH73, GH103, and CE4 target the glycosidic linkage of *N*-acetylmuramoyl or *N*-acetylglucosaminyl residues in peptidoglycan, which makes up a significant proportion of the chemical composition of coral mucus (29). GH16 enzymes are active on various algal structural polysaccharides including laminarin, agar, and carrageenan (30). Glycoside hydrolases from the GH3 family are exo-acting β-d-glucosidases, α-l-arabinofuranosidases, β-d-xylopyranosidases, *N*-acetyl-β-d-glucosaminidases, and *N*-acetyl-β-d-glucosaminide phosphorylases with broad substrate specificities that carry out a range of functions including plant and bacterial cell wall remodeling, cellulosic biomass degradation, and pathogen defense (31, 32). Our isolates revealed enriched GDEs known to target carbohydrates originating from algae and coral mucus. Notably, GDEs targeting plant cell wall carbohydrates in *Bacteroidetes* were found to be more abundant than those in other clades, while the frequency of sequences for GDEs targeting peptidoglycan was higher in *Proteobacteria* (Fig. 4B). Accordingly, we hypothesize distinct specializations among *Bacteroidetes* and *Proteobacteria* for their utilization of alga- and mucus-derived organic matter, respectively. Our results demonstrate that the specific potential for carbon metabolism facilitates the adaptation of the coral-associated bacteria to ecological niches with supplied carbohydrate substrates, and the carbohydrate composition of these microniches is likely to constitute one of the major driving forces that shape the local microbial community structures (33).

It is now well known that there are many genes involved in several intermediary metabolic pathways which tend to be reduced in endosymbiont genomes (34). In this study, we identified two of our isolates (SCSIO 12583 and SCSIO 12664) which had low GDE frequencies, for example. Low potential for carbohydrate utilization implies a relatively minor role in cycling reef organic carbon and preference for an obligate endosymbiotic lifestyle. Further evidence of this is the presence of *N*-acetyl-β-glucosaminidase in strain SCSIO 12664. As coral mucins could be *N*-glycosylated (29, 35), a bacterium with *N*-acetyl-β-glucosaminidase could therefore penetrate the host mucus layer (the coral’s first line of defense against invading bacteria [36]) and then subsequently establish endosymbiosis (35).

### Source of amino acids and polyamines in the coral holobiont.

The ability to synthesize amino acids varies among corals, with many essential amino acids acquired from Symbiodiniaceae and other exogenous sources (5, 37). The host genome in this study revealed that several amino acids could not be synthesized by the coral itself. Instead, *P. damicornis* may rely on its bacterial symbionts to assimilate these amino acids, in combination with its Symbiodiniaceae and heterotrophic feeding. Further, several isolates in this study were shown to potentially synthesize polyamines, such as putrescine and spermidine. Polyamines are involved in bacterial swarming motility and invasion ability (38, 39), biofilm formation and disassembly (40, 41), virulence (42–44), proper cell division (45, 46), and bacterial (47) and interkingdom (44) cell-cell communications. Interestingly, polyamines have also been found enriched in diseased coral colonies (48) and appear to play important roles in abiotic stress responses (49). At present, the relevance of polyamines produced by coral-associated bacteria to coral health is unknown though certainly worth further investigation. Finally, we were also able to identify several isolates which have the potential of biosynthesizing GABA from putrescine, and a *Labrenzia* strain (SCSIO 12622) which may be able to synthesize GABA from arginine. GABA shows multiple physiological functions, such as acting as an inhibitory neurotransmitter in the central nervous system of animals (50), gut modulation (51), protection against stress (52), and the regulation of settlement and metamorphosis of marine invertebrate larvae (53–55). Our results therefore highlight potential interactions mediated by bioactive compounds produced by these coral-associated bacteria.

### Putative opportunistic vibrio.

A previous meta-analytical study associated with culture collections of coral-associated bacteria identified that pathogenic and nonpathogenic *Vibrio* strains show functional separation based on their genome data (7). This offers a potentially useful tool to ascertain pathogenicity of members of this genus from genomic data and in the absence of laboratory inoculation trials. The functional profiles of the five vibrios cultured in this study demonstrated that they grouped with known pathogens (see Fig. S2 in the supplemental material), although the corals from which these bacteria were sampled showed no signs of compromised health. Interestingly, the abundant virulence factors present in these isolates also implied they are potentially opportunistic bacteria. But we still should interpret these findings with caution as the results are based only on the predicted functional profiles of these five vibrios, and further experiments are required for verifying their roles. Furthermore, we also found these five vibrios possessed multiple (between 45 and 60) putative antibiotic resistance genes, including *msbA*, *msrB*, *rpsJ*, and *tet* homologues (35). The true causal agents of the majority of coral diseases remain elusive (1, 56), though recently amoxicillin paste has been successfully used to treat corals affected by stony coral tissue loss disease (57, 58). The information about antibiotic resistance genes carried by the coral-associated bacteria obtained in this study may provide clues for the selection of antibiotics for both susceptible and resistant putative pathogens alongside potential beneficial communities.

**FIG 5:**
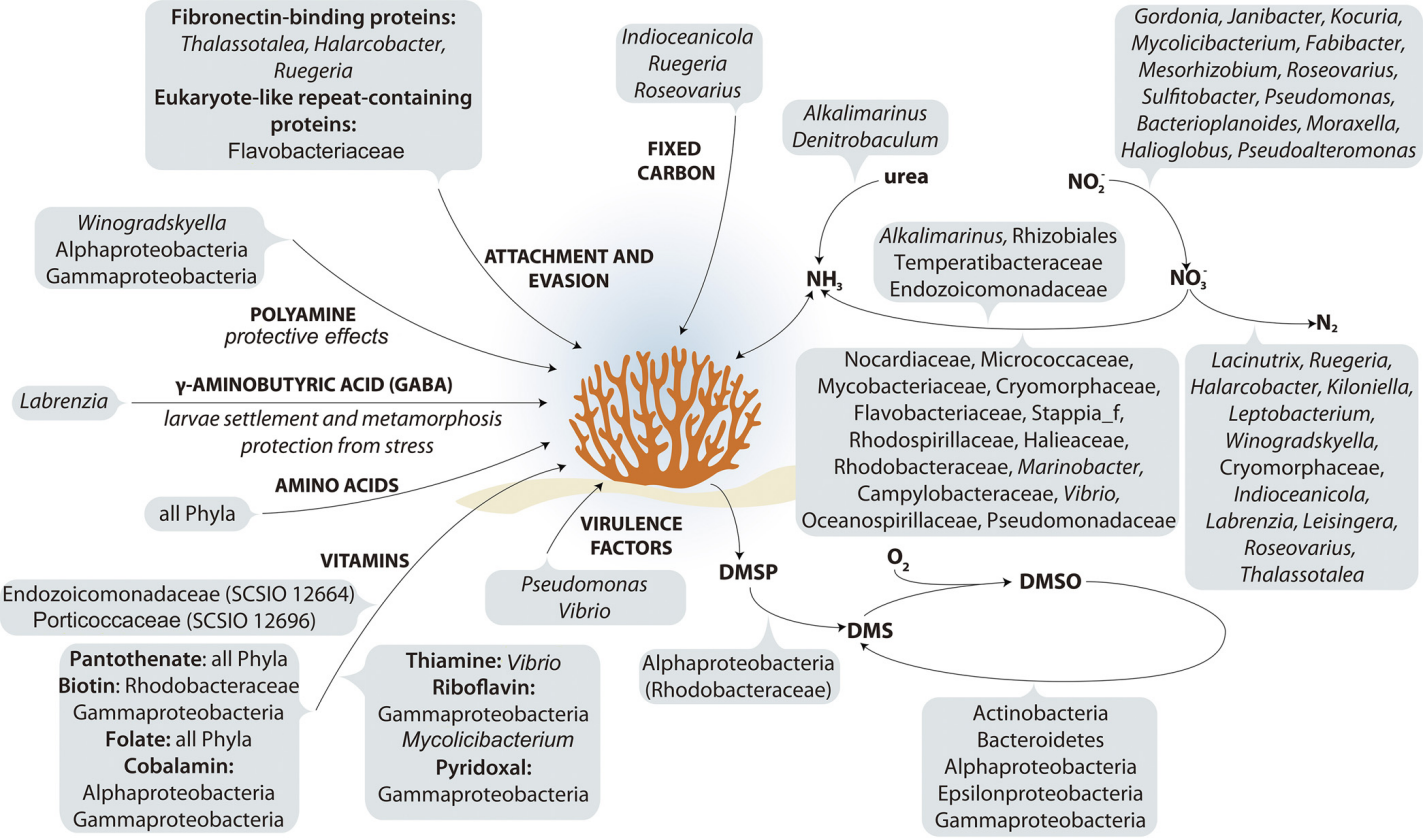
Schematic representing the proposed contributions of bacterial members to the *Pocillopora damicornis* holobiont.

To conclude, in this study we have increased the number of described cultured coral-associated bacteria by 395 isolates, 80 of which appear to be novel species and 17 from novel genera. Further, we sequenced 118 genomes of these isolates along with the host, *P. damicornis*. Although we recognize that this is not an exhaustive list of the cultural fraction from a coral, it likely represents the most in-depth study to date (accounting for ~8% of the total bacterial fraction of the host microbiome). That said, there are many taxa detected in 16S rRNA gene amplicon libraries which remain unculturable at this time, including many *Endozoicomonas* species (8). We did, however, culture a novel lineage of *Endozoicomonadaceae* (SCSIO 12664). This study therefore provides a strong base for future genetic studies that will improve the resolution of metagenomic analyses of the coral-associated microbiome. Moreover, pure cultures of the coral-associated microbiome acquired in this study will help to elucidate the role of bacteria in the coral holobiont (Fig. 5), assess the impact of specific isolates on coral health, and identify and provide beneficial microbes for efforts aimed at restoring reefs and improving coral health (3, 59).

## MATERIALS AND METHODS

### Coral-associated bacterial isolation.

*P. damicornis* samples were collected from the Luhuitou fringing reef at Sanya, China (109°28′E, 18°13′N), in December 2015, by scuba diving at a depth of 3 to 5 m. The mean seawater temperature, pH, and salinity for the sampling location in this month were 24.9°C, 7.9, and 33.6‰, respectively. No stressful environmental event was recorded 1 month prior to sample collection. Fragments of the coral colonies (measuring ca. 2 by 2 cm) were rinsed with sterile seawater to remove loosely attached bacteria and airbrushed to obtain a tissue slurry. Tissue slurries were prepared from four fragments cut from four individual genotypes (colonies separated by 0.5 m from each other), and the slurries were then pooled. A 1-mL aliquot of the slurry was then serially diluted 10^−1^, 10^−2^, 10^−3^, and 10^−4^, and each dilution was spread on separate plates containing marine agar 2216 (MA; Becton, Dickinson and Company [BD]). Petri dishes were incubated at 25°C, corresponding to the temperature of the ambient seawater, under aerobic conditions for 4 weeks. Single colonies were picked and streaked onto new plates to obtain single clones. All the isolates were stored in a glycerol suspension (20%, vol/vol) at −80°C and as freeze-dried cultures at 4°C.

### Bacterial identification via 16S rRNA gene sequence analysis.

Purified isolates were then subsequently recultivated on MA plates at the same temperature as above (25°C). Genomic DNA was extracted using the UltraClean microbial DNA isolation kit (Mo Bio Laboratories). The 16S rRNA gene was amplified using the bacterial universal primers 27F (5′-AGAGTTTGATCCTGGCTCAG-3′) and 1492R (5′-GGTTACCTTGTTACGACTT-3′), and the product was sequenced using the 3730*xl* DNA Analyzer (Applied Biosystems) with the software provided by the manufacturer. For the isolates in this study which had their genomes sequenced (see below), the 16S rRNA gene sequences were also extracted using RNAmmer (60), except for 10 isolates where extraction failed or the length of 16S rRNA gene sequences was less than those obtained through Sanger sequencing. Identification of phylogenetic neighbors and calculation of pairwise 16S rRNA gene sequence similarities were then achieved using EzBioCloud (http://www.ezbiocloud.net [61]). After multiple alignment of the data using Muscle, phylogenetic trees were generated using the neighbor-joining algorithms with MEGA X (62). Topologies of the phylogenetic trees were evaluated using the bootstrap resampling method of Felsenstein (63), for 1,000 resamplings. The output file was uploaded to iTOL for visualization (64). The species-level OTUs were clustered using mothur, with an identity of 98.7% as the species-level cutoff and an identity of 94.5% as the genus-level cutoff (65).

We then performed an alignment of these 16S rRNA gene sequences from the cultured bacteria using local BLAST and matched these to our previously published data set of 16S rRNA gene amplicon libraries from the same coral species at the same site (8). Here, we report the total number of aligned OTUs for each 16S rRNA gene sequence with the highest identity (E value of <3e−163, 100% coverage) in the 16S rRNA gene amplicon libraries.

### Coral-associated bacterial genome sequencing and processing.

One hundred eighteen bacterial genomes were sequenced as part of this study, 109 as “drafts” and nine classified as “complete.” The draft genomes were sequenced on the Illumina HiSeq 4000 system (Illumina) at the Beijing Genomics Institute (BGI; Shenzhen, China). Genomic DNA was sheared randomly to construct three read libraries with lengths of 270 bp by a Bioruptor ultrasonicator (Diagenode) and physicochemical methods. The paired-end fragment libraries were sequenced according to the Illumina HiSeq 4000 system protocol with 2- by 150-bp paired-end reads. Low-quality raw reads, with consecutive bases covered by fewer than five reads, were discarded. The sequenced reads were assembled using the SOAPdenovo 2.04 (66) software. Genome completeness was evaluated using CheckM v1.1.3 (67).

The complete genomes, in contrast, were sequenced on the PacBio RS platform and the Illumina HiSeq 4000 platform at the Beijing Genomics Institute (Shenzhen, China). Four Single Molecule, Real-Time (SMRT) cell zero-mode waveguide arrays of sequencing were used by the PacBio platform to generate the subread set. Subreads with lengths of <1 kb were removed. The program Pbdagcon was used for self-correction. Draft genome unitigs, which are uncontested groups of fragments, were assembled using the Celera Assembler against a high-quality corrected circular consensus sequence subread set. To improve the accuracy of the genome sequences, GATK (https://www.broadinstitute.org/gatk/) and SOAP tool packages (SOAP2, SOAPsnp, and SOAPindel) were used to make single-base corrections. To trace the presence of any plasmid, the filtered Illumina reads were mapped, using SOAP, to the bacterial plasmid database (http://www.ebi.ac.uk/genomes/plasmid.html).

Gene prediction was performed on the genome assemblies using glimmer3 (http://www.cbcb.umd.edu/software/glimmer/ [68]) with hidden Markov models. tRNA, rRNA, and small RNAs (sRNAs) were recognized using tRNAscan-SE (69), RNAmmer (60), and the Rfam database (70). Tandem repeat annotation was obtained using the Tandem Repeat Finder (71) (http://tandem.bu.edu/trf/trf.html), and the minisatellite DNA and microsatellite DNA were selected based on the number and length of repeat units.

The best hit for function annotation was retrieved using the BLAST alignment tool. Five databases, Kyoto Encyclopedia of Genes and Genomes (KEGG), Clusters of Orthologous Groups (COG), Non-Redundant Protein Database (NR), Swiss-Prot, and Gene Ontology (GO), were used for general function annotation. The genomes were also annotated against the carbohydrate-active enzymes (CAZy) database (www.cazy.org). The presence of signal peptides in carbohydrate-active enzymes was predicted using the SignalP 5.0 server (72). The genome sequences were mapped against KEGG modules by using the reconstruct pathway in KEGG Mapper available at the KEGG website (73). Virulence factors and antibiotic resistance genes were annotated using the Virulence Factors Database (VFDB [74]) and the Comprehensive Antibiotic Resistance Database (CARD [75]), respectively.

The isolates, which were considered candidate novel taxa according to the threshold of 98.7% 16S rRNA gene sequence identity, were further analyzed with the digital DNA-DNA hybridization (dDDH) value, average nucleotide identity (ANI), and the percentage of conserved proteins (POCP) based on their genomes. The dDDH value was calculated using the online Genome to Genome Distance Calculator (GGDC 2.1; http://ggdc.dsmz.de [76]). The ANI of genome to genome was calculated using JSpeciesWS (77). The POCP was calculated using BLASTp (78). The phylogenomic tree was constructed using GTDB-Tk (79).

### Coral genome sequencing and processing.

For investigating putative metabolic lineages between coral host and its bacterial symbionts, we also sequenced the genome of *P. damicornis*. A single colony of *P. damicornis* was collected from the same reef (Luhuitou fringing reef) as the microbial analysis (above) but kept in an indoor aquarium with circulating artificial seawater (Reef Crystals sea salt [Aquarium Systems]). To minimize endosymbiotic Symbiodiniaceae DNA contamination, six nubbins (approximately 3 cm high, 1- cm diameter) were cut from the colony, subjected to heat stress to induce bleaching, and then used for DNA extraction. Briefly, the water temperature was increased gradually (1°C per day) from 25 to 32°C and then maintained at 32°C for 7 days.

Coral tissues from the bleached nubbins were then removed from the skeleton using 0.2-μm-filtered autoclaved artificial seawater that was sprayed using a syringe. Tissue suspensions were then centrifuged at 2,000 × *g* for 10 min (4°C), and DNA was extracted from the cell pellet using the Qiagen genomic DNA kit, according to the manufacturer’s instructions. DNA quantity and quality were assessed by NanoDrop, Qubit fluorometer, and 0.5% agarose gel electrophoresis.

The library with an insert size of 300 to 400 bp was paired-end sequenced using DNBSEQ (Beijing Genomics Institute Technology [BGI Tech]), ultimately resulting in 142.84-Gb clean reads. The sequencing library with 20-kb DNA inserts was constructed and then sequenced using the PacBio Sequel platform (Pacific Biosciences), resulting in 110.40-Gb subreads. The size of the *P. damicornis* genome was estimated by *k*-mer distribution analysis to be 382 Mb. The PacBio data were *de novo* assembled using Falcon (https://github.com/PacificBiosciences/FALCON [80]). Sequencing errors in assembled sequences were corrected with the Arrow program using PacBio raw data. We also used DNBSEQ data to improve the assembly result by Pilon (81). In addition, the genome assembly completeness was assessed by using BUSCO (82) (metazoa_odb10). We also validated the assembled genome using 12,188 unigenes (length ≥300 bp) from transcriptome sequencing (RNA-Seq) (8). The contig *N*_50_ of the assembled genome is 3 Mb, and the completeness is 94.2%.

*De novo*, transcriptome-based and homologue-based approaches were combined to predict genes in the assembled genome. Protein databases of Acropora millepora (GCF_004143615.1), Dendronephthya gigantea (GCF_004324835.1_DenGig_1.0), Nematostella vectensis (GCF_000209225.1_ASM20922v1), and *P. damicornis* (GCF_003704095.1_ASM370409v1) were used as references in the homologue-based gene prediction by Genewise v2.4.1 software. Subsequently, the NR, Swiss-Prot, TrEMBL, KEGG, InterPro, and GO databases were used for further annotation of protein-coding genes using BLAST v2.2.31.

### Polyphasic taxonomy analyses of bacterial isolates.

Nine isolates, representing the most potentially novel of our isolated taxa and/or those which were deemed to have high relevance for coral-associated bacteria (such as members in the *Endozoicomonadaceae* and *Cellvibrionales*) were subjected to polyphasic taxonomy. Gram staining was performed using the Gram stain kit (Guangdong HuanKai Microbial Sci. & Tech, Co., Ltd.). The isolates were then imaged under a transmission electron microscope (Hitachi TEM system HC-1) to give greater differentiation on cellular morphology. Finally, cell motility was also confirmed by the development of turbidity throughout a tube containing semisolid (0.3% [wt/vol] agar) MA medium.

Growth of these nine isolates was assessed under anaerobic conditions and evaluated on an MA plate in the Whitley DG250 Anaerobic Workstation (Don Whitley Scientific, England). The requirement of seawater for growth was assessed using both MA and Trypticase soy agar (TSA; Becton, Dickinson and Co.). For the TSA, artificial seawater was initially utilized and then modified with 3% (wt/vol) NaCl. Growth at different temperatures was also assessed (5, 10, 15, 20, 25, 30, 33, 35, 37, 40, 42, and 45°C), and growth at different NaCl concentrations was measured by incubating the cultures in TSA (0.5 to 20% NaCl, wt/vol) or MA (3 to 20% NaCl, wt/vol) for 6 days at 25 or 30°C. Growth at different pHs (pH 4, 5, 6, 7, 8, 9, and 10, using the buffer system described by Xu et al. [83]) was measured by culturing the isolates in marine broth (MB; Becton, Dickinson and Co.) for 6 days at 25 or 30°C. Physiological and biochemical tests were performed using the API ZYM system (bioMérieux) and Biolog GEN III microplate (Biolog Inc.).

These nine isolates also underwent chemotaxonomic classification. The bacterial cells used for chemotaxonomic analysis were obtained from cultures grown in MB medium at 25 or 30°C and 180 rpm for 48 to 72 h. The respiratory quinones were extracted and purified as described by Collins et al. (84) and analyzed using high-pressure liquid chromatography (HPLC) (85). Polar lipids were also examined via two-dimensional thin-layer chromatography (TLC) and identified as per references 86 and 87. Cellular fatty acids were extracted, methylated, and analyzed using the Sherlock microbial identification system (MIDI, Inc.), and profiles were identified using Sherlock version 6.2B (MIDI database: TSBA6).

### Vitamin assay.

Results indicated that strain SCSIO 12664 belonged to the *Endozoicomonadaceae*, a likely endosymbiont of coral (22, 23), and strain SCSIO 12696 belonged to the *Cellvibrionales* with a relatively small genome (2.93 Mb), indicating it might be a potential endosymbiont. Therefore, production of vitamins was assessed for these two isolates (SCSIO 12664 and SCSIO 12696) which were both originally grown in MB at 25°C and 30°C, respectively. Culture broth was centrifuged (10,000 × *g*, 10 min, 4°C), and the supernatant was filtered with a Millipore 0.22-μm GSWP membrane filter (Merck) and then used for extracellular vitamin analysis. Pellets were collected and washed with filtered and autoclaved artificial seawater, which was prepared using sea salts (Sigma-Aldrich), for intracellular vitamin analysis. Cyanocobalamin was determined using the VitaFast quantification kit (R-Biopharm AG, Germany). Thiamine, riboflavin, and pyridoxine were determined as described by Keuth and Bisping (88). Folic acid, biotin, and pantothenic acid were determined by means of microbiological assays according to the methods of Hugenschmidt et al. (89), Han et al. (90), and Tsuda et al. (91), respectively. Lactobacillus rhamnosus (ATCC 7469) was used for the folic acid assay, and Lactobacillus plantarum (ATCC 8014) was used to determine the contents of biotin and pantothenic acid.

### Data availability.

Sequence data generated in this study are available under NCBI BioProject numbers PRJNA723412 and PRJNA725659 and GenBank accession numbers MZ262762 to MZ263174.
