# Supplementary figures and images for: Cultured Bacteria Provide Insight into the Functional Potential of the Coral-Associated Microbiome
Source: mSystems. 2022 Jun 13;7(4):e00327-22. doi: 10.1128/msystems.00327-22 (PMC9426491; doi:10.1128/msystems.00327-22)

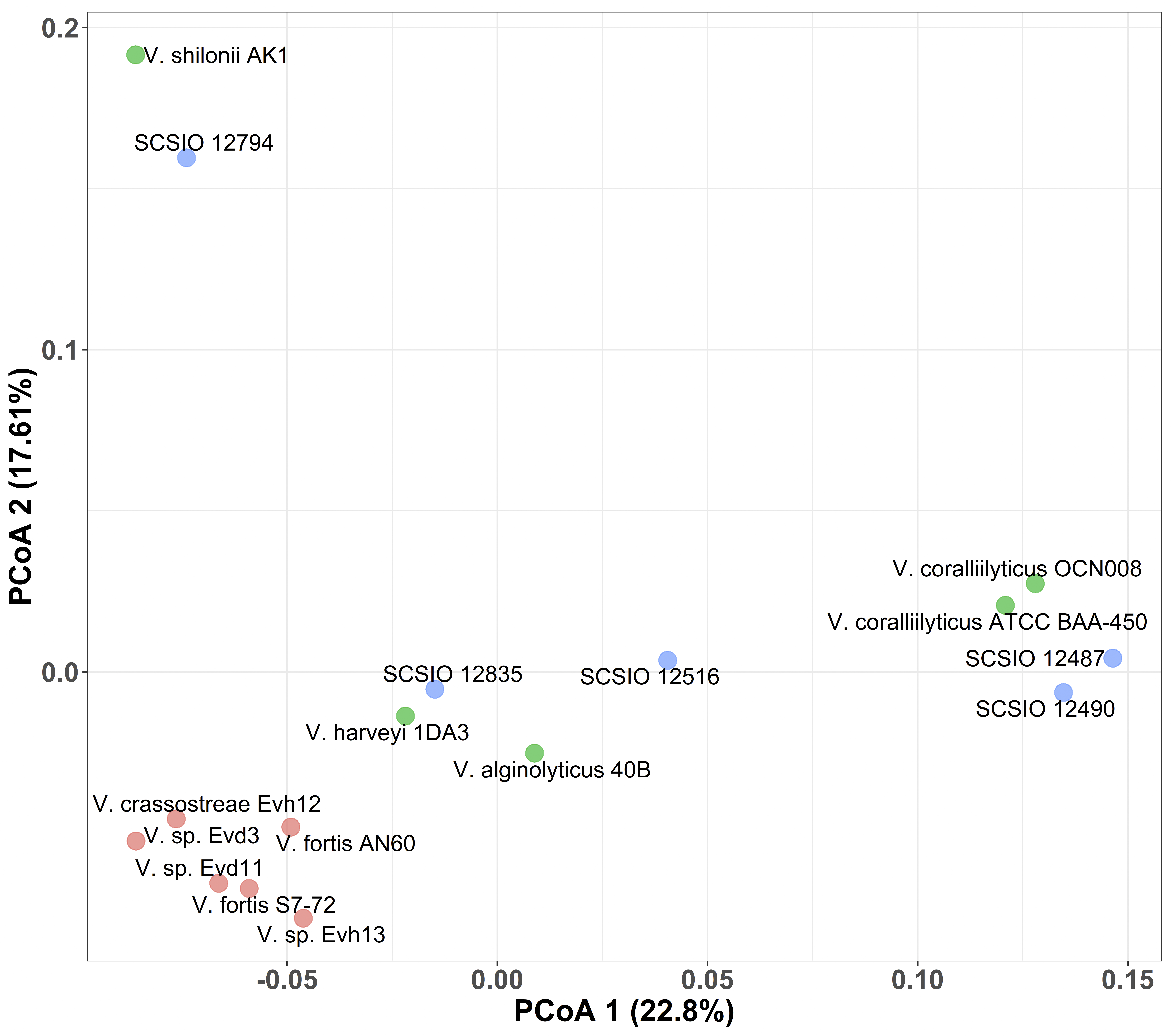

Supplement: FIG S2 [file msystems.00327-22-sf002.tif]

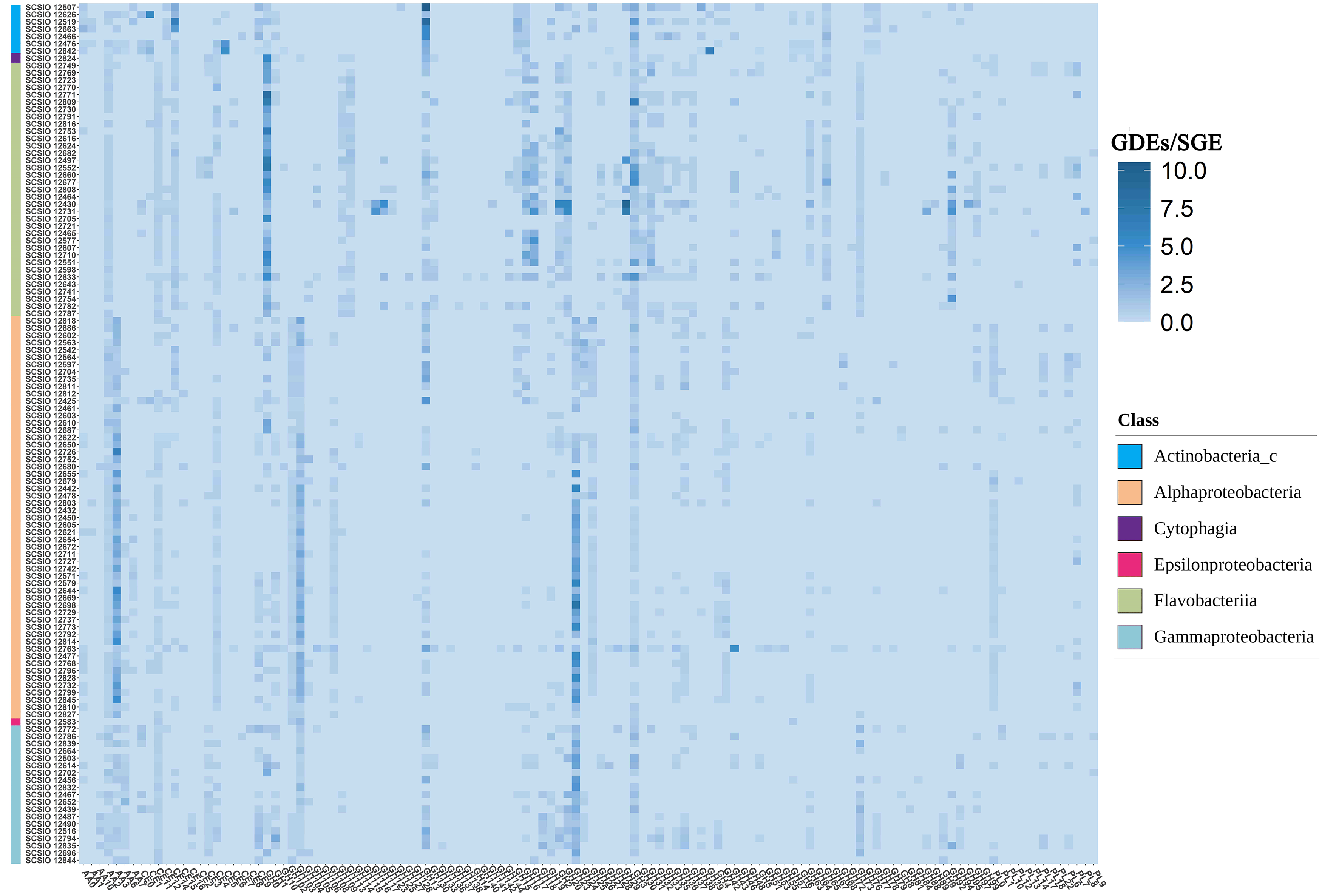

Supplement: FIG S3 [file msystems.00327-22-sf003.tif]
