# Supplementary material for: Cultured Bacteria Provide Insight into the Functional Potential of the Coral-Associated Microbiome
Source: mSystems. 2022 Jun 13;7(4):e00327-22. doi: 10.1128/msystems.00327-22 (PMC9426491; doi:10.1128/msystems.00327-22)
Supplement: DATA SET S1 [file msystems.00327-22-sd001.pdf]

Data S1. Description of novel taxa

Description of *Bacterioplanoides vesiculosa* sp. nov.

Bacterioplanoides vesiculosa (ve.si.cu.lo'sa L. fem. adj. *vesiculosa*, full of blisters, vesiculous).

The closest phylogenetic neighbour is *Bacterioplanoides pacificum*, which shares a 16S rRNA gene sequence identity of 98.49% (Fig. 1). Although we can not compare genomes of the strain SCSIO 12839^T and *Bacterioplanoides pacificum* as there is no genome information of *Bacterioplanoides pacificum* in public datasets, the result of the phylogenetic analysis according to 16S rRNA gene sequences supports the separate species status. The dDDH and ANI values were <33.6% and 75.45%, respectively, between the genomes of SCSIO 12839^T and other relatives.

Cells are Gram-stain-negative, aerobic, motile by means of a single polar flagellum, curved rod or spiral-shaped (Fig. 2). Colonies are circular, creamy white, smooth with entire margins and 1.0 mm in diameter on marine agar after cultivation for 3 days at 25 °C. Cells do not growth without seawater. Growth occurs at 15–37 °C (optimum, 25–30 °C), at pH 6–8 and with 3–7% (w/v) NaCl concentrations. Oxidase is positive, but catalase is negative. Negative for hydrolysis of gelatin and starch, nitrate reduction, and urease activity. Positive for esterase lipase (C8), valine arylamidase, cystine arylamidase, trypsin, acid phosphatase, and naphthol-AS-BI-phosphohydrotase, but negative for *β*-fucosidase, N-acetyl-*β*-glucosaminidase, alkaline phosphatase, esterase (C4), *α*-galactosidase, *β*-galactosidase, leucine arylamidase, *α*-glucosidase, *β*-glucosidase, lipase (C14),

α-chymotrypsin, α-mannosidase, and β-glucuronidase. Dextrin, D-trehalose, D-cellobiose, gentiobiose, sucrose, D-turanose, stachyose, D-raffinose, α-D-lactose, D-melibiose, D-salicin, N-acetyl-D-glucosamine, N-acetyl-β-D-mannosamine, N-acetyl-D-galactosamine, α-D-glucose, D-mannose, D-fructose, D-galactose, 3-methyl glucose, D-fucose, L-fucose, L-rhamnose, inosine, D-sorbitol, D-mannitol, D-arabitol, myo-inositol, D-glucose-6-PO_4, D-fructose-6-PO_4, α-keto-glutaric acid, pectin, D-galacturonic acid, L-galactonic acid lactone, glucuronamide, D-saccharic acid, citric acid, tween 40, acetoacetic acid, propionic acid, L-lactic acid, and acetic acid are utilized as sole carbon and energy sources but D-maltose, β-methyl-D-glucoside, glycerol, L-aspartic acid, L-malic acid, quinic acid, D-glucuronic acid, N-acetyl neuraminic acid, L-arginine, mucic acid, gelatin, L-serine, D-aspartic acid, D-malic acid, L-glutamic acid, L-pyroglutamic acid, glycyl-L-prolin, L-histidine, D-serine, L-alanine, methyl pyruvate, D-gluconic acid, *p*-hydroxy-phenylacetic acid, D-lactic acid methyl ester, α-keto-butyric acid, α-hydroxy-butyric acid, bromo-succinic acid, formic acid, γ-amino-butryric acid, and β-hydroxy-D,L butyric acid are not. Inhibited by guanidine HCl, but not by niaproof 4, nalidixic acid, potassium tellurite, sodium butyrate, sodium bromate, lithium chloride, 1% sodium lactate, fusidic acid, minocycline, troleandomycin, rifamycin SV, lincomycin, tetrazolium violet, tetrazolium blue, vancomycin, and aztreonam. The main cellular fatty acids (>5%) are summed feature 3 ($C_{16:1}\omega 7c$/$C_{16:1}\omega 6c$), $C_{16:0}$, iso-$C_{16:0}$, and summed feature 8 ($C_{18:1}\omega 7c$/$C_{18:1}\omega 6c$). The respiratory quinone is Q-9. The polar lipids present are diphosphatidylglycerol, phosphatidylethanolamine, one unidentified aminolipid, and

three unidentified lipids (Fig. 3). The G+C content of the genomic DNA is 47.92%. The type strain is SCSIO 12839^{T}.

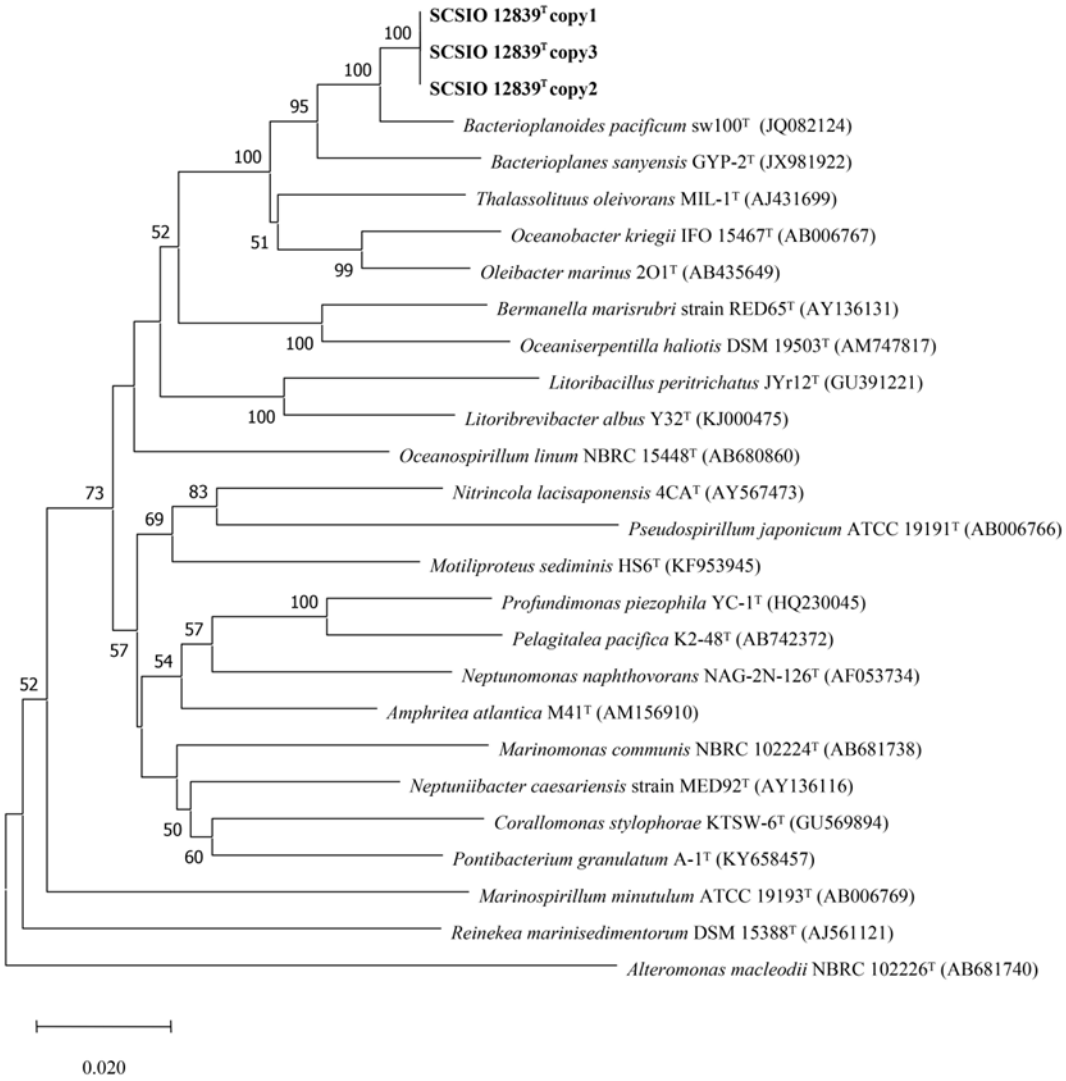

Figure 1. Neighbour-joining phylogenetic tree reconstructed based on 16S rRNA gene sequences, showing the position of strain SCSIO 12839^{T} and other closely related species. Three 16S rRNA gene copies of strain SCSIO 12839^{T} were included in phylogenetic analysis. Only bootstrap values (percentages of 1000 replications) >50% were shown. The strain characterized in this study is shown in bold type. All ambiguous positions were removed for each sequence pair (pairwise deletion option). There were a total of 1592 positions in the final dataset. Bar, 0.02 changes per nucleotide position.

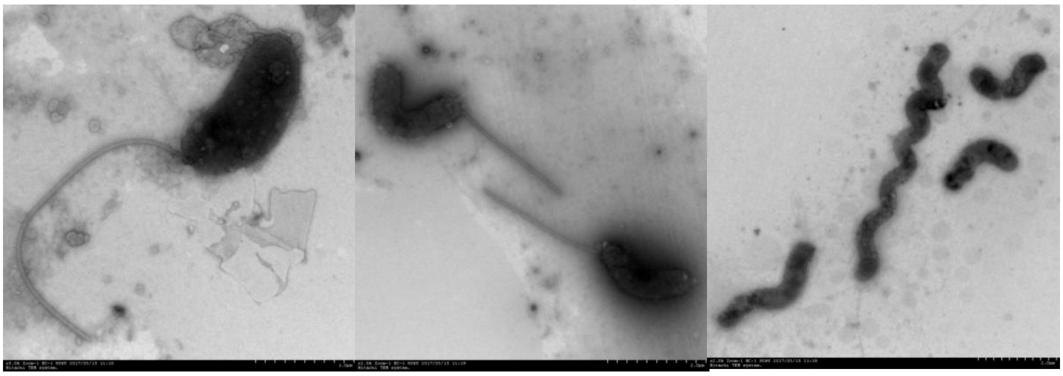

Figure 2. Transmission electron microscope photographs of strain SCSIO 12839^{T}.

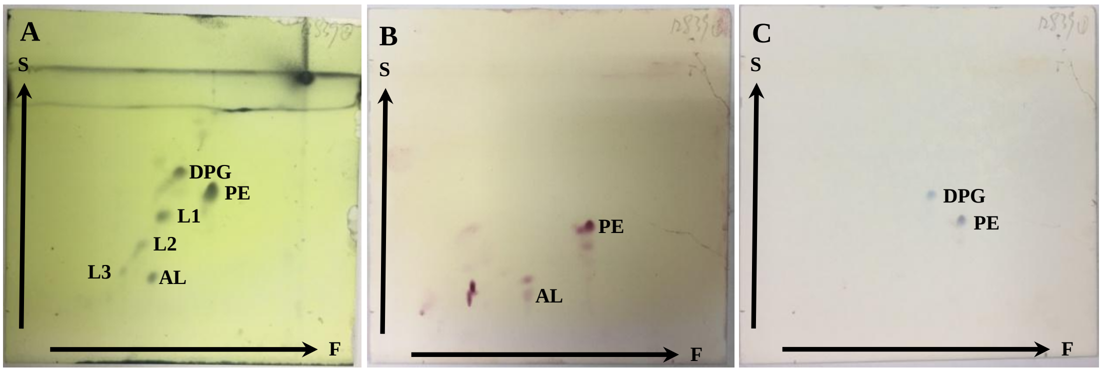

Figure 3. Two-dimensional thin layer chromatogram of polar lipids of strain SCSIO 12839^{T}. Chromatograms were run as follows: Silica Gel 60 thin-layer plates (10 by 10 cm) were spotted with 10 µL of a whole-cell lipid extract. Chloroform/methanol/water (65:25:4, by vol.) was used in the first direction, and chloroform/acetic acid/methanol/water (80:18:12:5, by vol.) was used in the second direction. A, The plate was sprayed with molybdatophosphoric acid; B, The plate was sprayed with ninhydrin reagent; C, The plate was sprayed with molybdenum blue reagent; DPG, diphosphatidylglycerol; PE, phosphatidylethanolamine; AL, unidentified aminolipid; L, unidentified polar lipid. F, first dimension of thin layer chromatogram; S, second dimension of thin layer chromatogram.

Description of *Corallivibrio* gen. nov.

Corallivibrio (Co.ral.li.vi'bri.o L. neut. n. *corallium*, coral; N.L. masc. n. *vibrio*, a vibrio; N.L. masc. n. *Corallivibrio*, a vibrio from the coral).

Strain SCSIO 12827^{T} showed the highest 16S rRNA gene similarity (90.69%) to *Methyloligella solikamskensis*. While according to 16S rRNA gene-based phylogeny, the strain is placed at a distinct clade from type species within type genera of families in Rhizobiales (Fig. 4). The highest POCP value of 37.52% also separates it

from other genera.

Gram-stain-negative, aerobic, curved or rod-shaped, motile, having a single polar flagellum, or a single flagellum at each end (Fig. 5). Oxidase-positive, catalase-negative. The main cellular fatty acids are summed feature 8 ($C_{18:1}\omega 7c$/$C_{18:1}\omega 6c$), summed feature 3 ($C_{16:1}\omega 7c$/$C_{16:1}\omega 6c$), and $C_{16:0}$. The major polar lipids are phosphatidylglycerol and phosphatidylethanolamine (Fig. 6). The G+C content of genomic DNA of the type strain is 62.98%. The type species is *Corallivibrio flagellatus*.

Description of *Corallivibrio flagellatus* sp. nov.

Corallivibrio flagellatus (fla.gel.la'tus L. neut. n. *flagellum*, a whip; L. masc. adj. suff. –*atus*, suffix denoting provided with; L. masc. part. adj. *flagellatus*, flagellated).

In addition to the properties given in the genus description, colonies on marine agar are creamy white, smooth and circular with regular margins. The colony size is approximately 0.5 mm in diameter after 2 days at 25 °C. Positive for hydrolysis of starch and gelatin. Negative for urease activity and nitrate reduction. Growth occurs at 20–42 °C (optimum, 30 °C), at pH 5–7 and with 3–7% (w/v) NaCl concentrations. Positive for alkaline phosphatase, esterase (C4), esterase lipase (C8), lipase (C14), leucine arylamidase, valine arylamidase, cystine arylamidase, trypsin, *α*-chymotrypsin, acid phosphatase, *α*-galactosidase, *β*-galactosidase, *α*-glucosidase, *β*-glucosidase, *α*-mannosidase, and naphthol-AS-BI-phosphohydrotase, but negative for *β*-fucosidase, N-acetyl-*β*-glucosaminidase, and *β*-glucuronidase. Dextrin, D-maltose, D-cellobiose, gentiobiose, sucrose, D-turanose, stachyose, D-raffinose,

α-D-lactose, D-melibiose, D-salicin, *α*-D-glucose, D-mannose, D-fructose, D-galactose, 3-methyl glucose, D-fucose, L-fucose, L-rhamnose, inosine, D-sorbitol, D-mannitol, D-arabitol, myo-inositol, glycerol, D-fructose-6-PO_4, L-aspartic acid, L-histidine, glucuronamide, mucic acid, quinic acid, D-saccharic acid, citric acid, acetoacetic acid, propionic acid, and acetic acid are utilized as sole carbon and energy sources but D-trehalose, *β*-methyl-D-glucoside, N-acetyl-*β*-D-mannosamine, N-acetyl-D-glucosamine, N-acetyl-D-galactosamine, D-glucose-6-PO_4, N-acetyl neuraminic acid, pectin, L-arginine, L-galactonic acid lactone, D-glucuronic acid, gelatin, L-serine, D-aspartic acid, L-glutamic acid, D-galacturonic acid, L-pyroglutamic acid, D-serine, L-alanine, glycyl-L-prolin, methyl pyruvate, *α*-keto-glutaric acid, D-gluconic acid, D-malic acid, L-malic acid, *α*-keto-butyric acid, *p*-hydroxy-phenylacetic acid, D-lactic acid methyl ester, *α*-hydroxy-butyric acid, L-lactic acid, bromo-succinic acid, formic acid, tween 40, *γ*-amino-butryric acid, and *β*-hydroxy-D,L butyric acid are not. Inhibited by troleandomycin, guanidine HCl, and lithium chloride but not by fusidic acid, 1% sodium lactate, minocycline, rifamycin SV, lincomycin, tetrazolium violet, tetrazolium blue, vancomycin, nalidixic acid, potassium tellurite, aztreonam, niaproof 4, sodium butyrate, and sodium bromate. The type strain is SCSIO 12827^T.

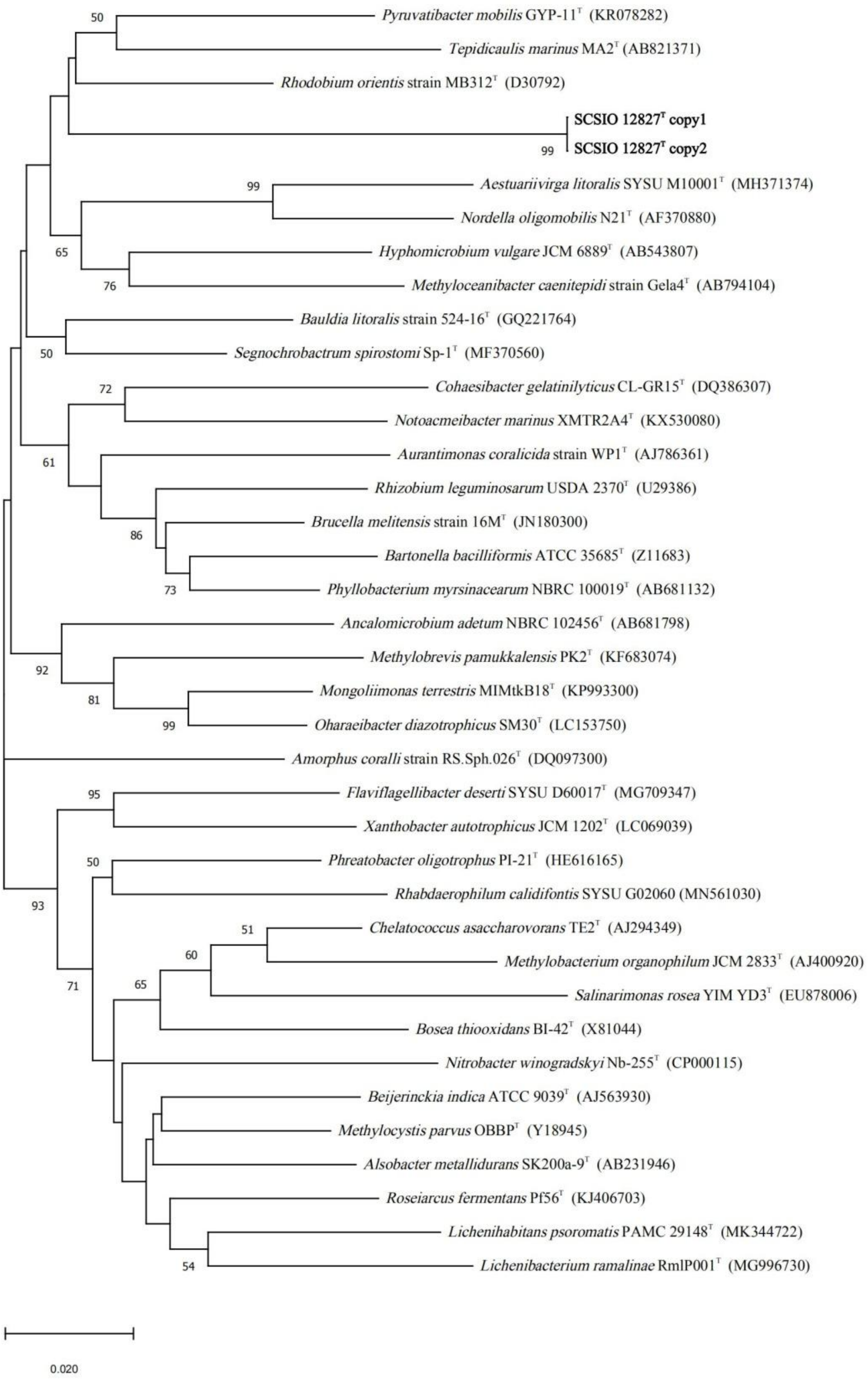

Figure 4. Neighbour-joining phylogenetic tree reconstructed based on 16S rRNA gene sequences, showing the position of strains SCSIO 12827^{T} and other type species within type genera of families in the order Rhizobiales. Two 16S rRNA gene copies of strain SCSIO 12827^{T} were included in phylogenetic analysis. Only bootstrap values (percentages of 1000 replications) >50% were shown. The strain characterized in this study is shown in bold type. All ambiguous positions were removed for each sequence pair (pairwise deletion option). There were a total of 1557 positions in the final dataset. Bar, 0.02 changes per nucleotide position.

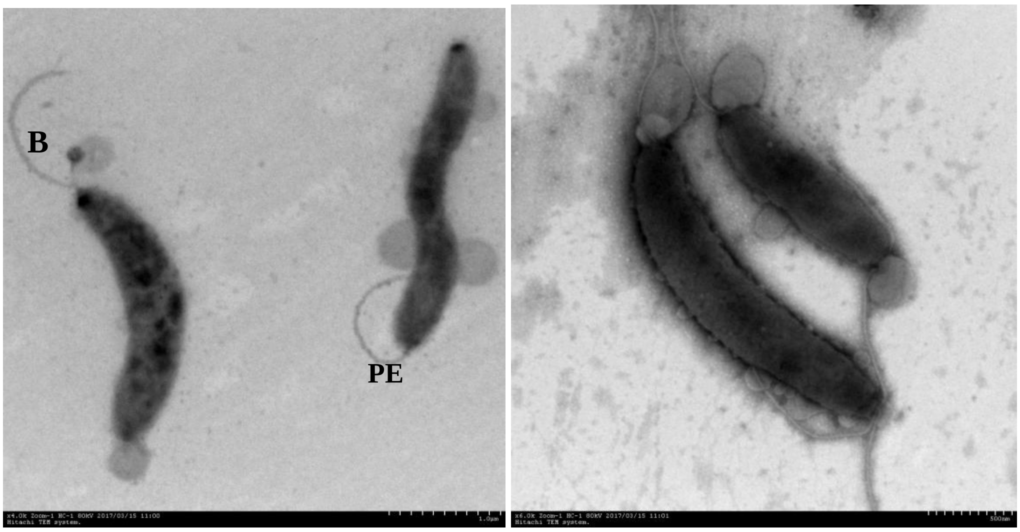

Figure 5. Transmission electron microscope photographs of strain SCSIO 12827^T.

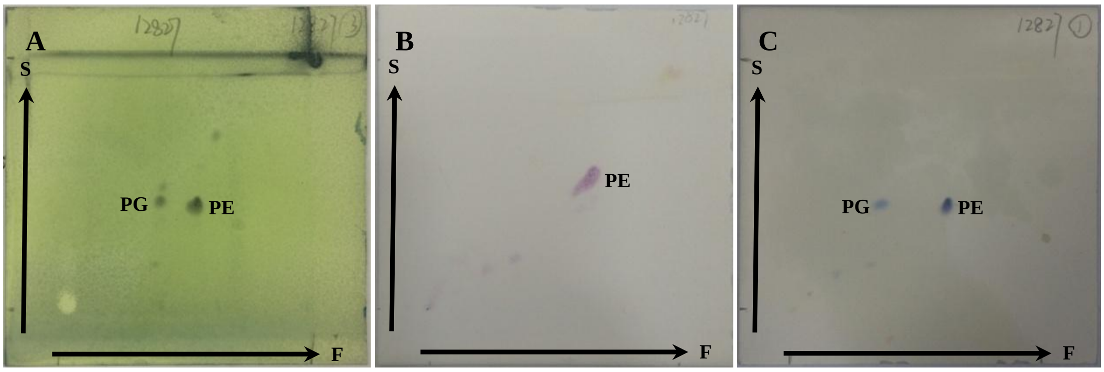

Figure 6. Two-dimensional thin layer chromatogram of polar lipids of strain SCSIO 12827^T. Chromatograms were run as follows: Silica Gel 60 thin-layer plates (10 by 10 cm) were spotted with 10 µL of a whole-cell lipid extract. Chloroform/methanol/water (65:25:4, by vol.) was used in the first direction, and chloroform/acetic acid/methanol/water (80:18:12:5, by vol.) was used in the second direction. A, The plate was sprayed with molybdatophosphoric acid; B, The plate was sprayed with ninhydrin reagent; C, The plate was sprayed with molybdenum blue reagent; PG, phosphatidylglycerol; PE, phosphatidylethanolamine. F, first dimension of thin layer chromatogram; S, second dimension of thin layer chromatogram.

Description of *Corallivirga* gen. nov.

Corallivirga (Co.ral.li.vir'ga L. neut. n. *corallium*, coral; L. fem. n. *virga*, rod; N.L. fem. n. *Corallivirga*, a rod from a coral, referring to the isolation of the first strains from the coral *Pocillopora damicornis*).

Phylogenetically, strain SCSIO 12696^T is affiliated to the family Porticoccaceae, and forms a phyletic lineage that is distinct from the known genus *Porticoccus* within Porticoccaceae (Fig. 7). The POCP, ANI, and dDDH values between SCSIO 12696^T and "*Porticoccus hydrocarbonoclasticus"* (no genome information of the type species *Porticoccus litoralis*) are 50.97%, 67.42%, and 21.7%, respectively. Although the POCP value is nearing the genus-level threshold of 50%, the 16S rRNA gene sequence identity is 91.83% and 91.18% to *Porticoccus litoralis* and "*Porticoccus hydrocarbonoclasticus"*, respectively, which is markedly below established genera delineation thresholds. Thus, we propose it represents a novel genus.

Gram-stain-negative, aerobic, oxidase-positive and catalase-negative. Cells are rod or oval-shaped, single or in pairs, have a single polar or lateral flagellum (Fig. 8). Requiring NaCl for growth. Ubiquinone Q-10 is the major respiratory quinone. Predominant cellular fatty acids are iso-$C_{15:0}$, iso-$C_{13:0}$, anteiso-$C_{15:0}$, iso-$C_{17:0}$, and iso-$C_{11:0}$ 3-OH. The polar lipids present are diphosphatidylglycerol, phosphatidylethanolamine, phosphatidylglycerol, and an unknown phospholipid (Fig. 9). The G+C content of the genomic DNA of the type strain is 51.98%. The type species is *Corallivirga polyvitaminum*.

Description of *Corallivirga polyvitaminum* sp. nov.

Corallivirga polyvitaminum (po.ly.vi.ta.mi'nu.m Gr. adj. *poly*, many; N.L. neut. n. *vitaminum*, vitamin; N.L. neut. n. *polyvitaminum*, related to many vitamins, referring to the ability to produce numerous vitamins).

In addition to the properties given in the genus description, positive for hydrolysis of starch, negative for hydrolysis of gelatin, urease activity, and nitrate reduction. Growth occurs at 10–35 °C (optimum, 30-35 °C), at pH 5–7 and with 3–7% (w/v) NaCl concentrations (optimum, 3%). Positive for alkaline phosphatase, esterase (C4), esterase lipase (C8), lipase (C14), leucine arylamidase, valine arylamidase, cystine arylamidase, trypsin, *α*-chymotrypsin, acid phosphatase, *α*-glucosidase, *β*-glucosidase, and naphthol-AS-BI-phosphohydrotase, but negative for *α*-mannosidase, *β*-fucosidase, *α*-galactosidase, *β*-galactosidase, N-acetyl-*β*-glucosaminidase, and *β*-glucuronidase. Dextrin, D-maltose, D-trehalose, D-cellobiose, gentiobiose, sucrose, D-turanose, stachyose, D-raffinose, *α*-D-lactose, D-melibiose, *β*-methyl-D-glucoside, D-salicin, N-acetyl-*β*-D-mannosamine, *α*-D-glucose, D-fructose, D-mannose, N-acetyl-D-glucosamine, N-acetyl-D-galactosamine, D-galactose, 3-methyl glucose, D-fucose, L-fucose, L-rhamnose, inosine, D-sorbitol, D-mannitol, D-arabitol, myo-inositol, D-glucose-6-PO_4, D-fructose-6-PO_4, L-histidine, pectin, L-galactonic acid lactone, D-glucuronic acid, glucuronamide, quinic acid, D-saccharic acid, citric acid, D-malic acid, L-malic acid, *α*-keto-butyric acid, acetoacetic acid, propionic acid, formic acid, and acetic acid are utilized as sole carbon and energy sources but N-acetyl neuraminic acid, L-arginine, L-aspartic acid, glycerol, gelatin, L-serine, D-aspartic acid, L-glutamic acid, D-galacturonic acid, L-pyroglutamic acid, mucic acid, D-serine, L-alanine, glycyl-L-prolin, methyl pyruvate, *α*-keto-glutaric acid, D-gluconic acid, *p*-hydroxy-phenylacetic acid, D-lactic acid methyl ester, *α*-hydroxy-butyric acid,

L-lactic acid, bromo-succinic acid, tween 40, *γ*-amino-butryric acid, and *β*-hydroxy-D,L butyric acid are not. Inhibited by 1% sodium lactate, troleandomycin, guanidine HCl, vancomycin, nalidixic acid, and lithium chloride but not by fusidic acid, minocycline, rifamycin SV, lincomycin, tetrazolium violet, tetrazolium blue, potassium tellurite, aztreonam, niaproof 4, sodium butyrate and sodium bromate. The type strain is SCSIO 12696^{T}.

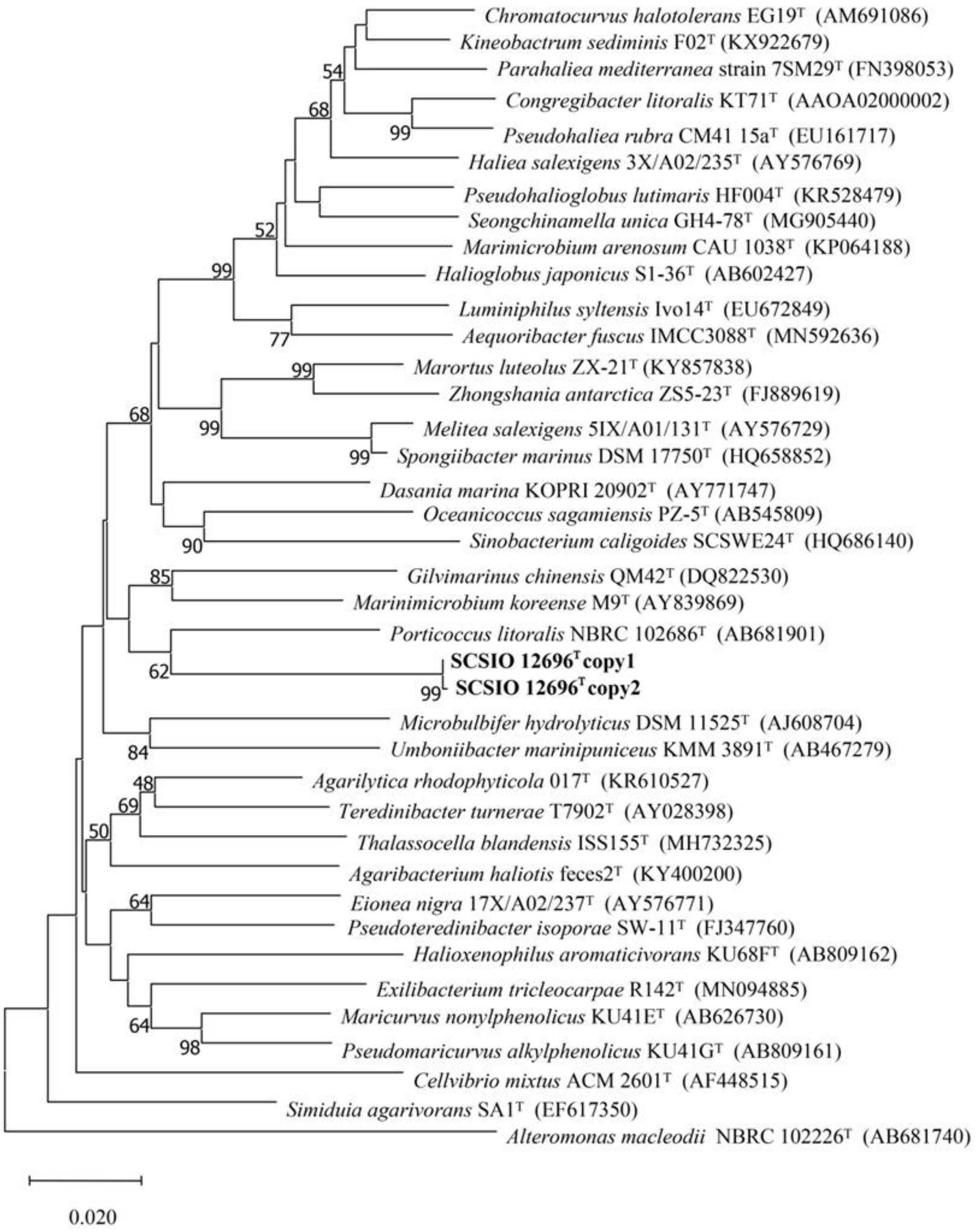

Figure 7. Neighbour-joining phylogenetic tree reconstructed based on 16S rRNA gene sequences, showing the position of strain SCSIO 12696^{T} and other closely related species. Two 16S rRNA gene copies of strain SCSIO 12696^{T} were included in phylogenetic analysis. Only bootstrap values (percentages of 1000 replications) >50% were shown. The strain characterized in this study is shown in bold type. All ambiguous positions were removed for each sequence pair (pairwise deletion option). There were a total of 1567 positions in the final dataset. Bar, 0.02 changes per nucleotide position.

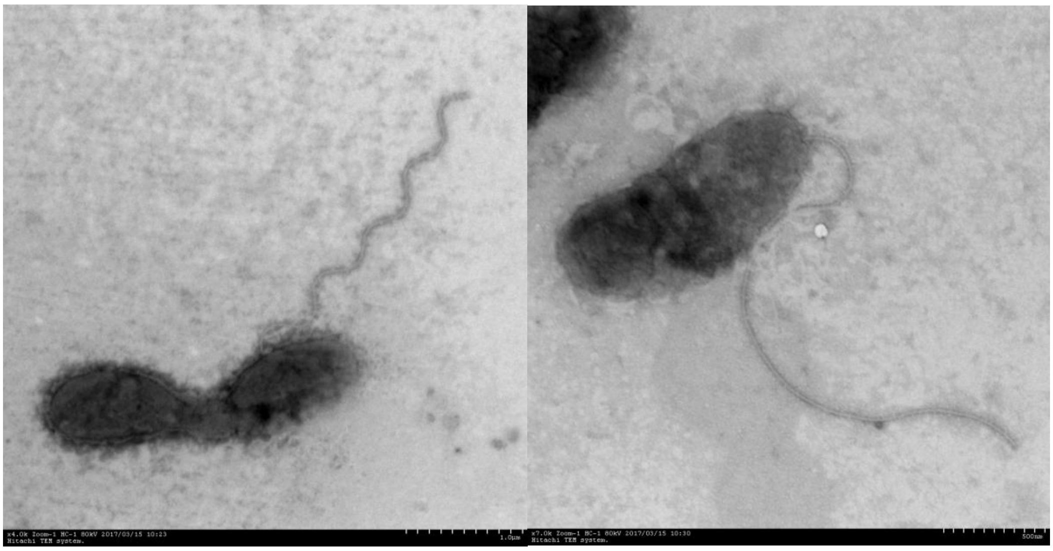

Figure 8. Transmission electron microscope photographs of strain SCSIO 12696^{T}.

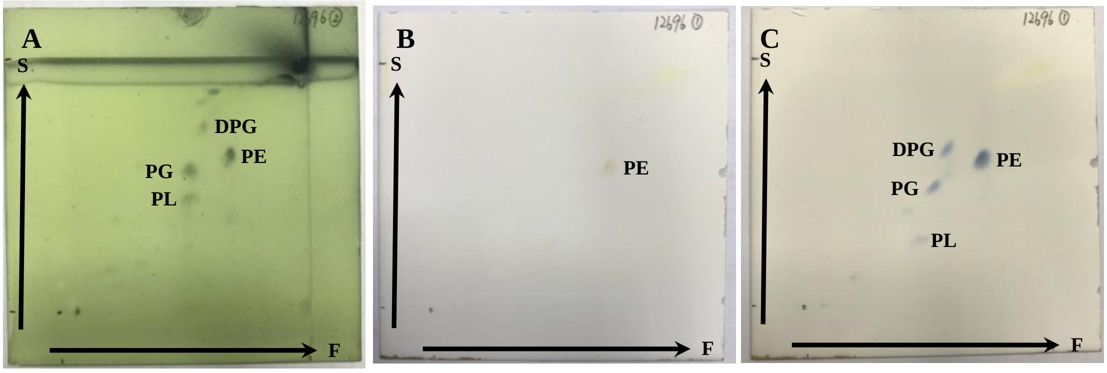

Figure 9. Two-dimensional thin layer chromatogram of polar lipids of strain SCSIO 12696^{T}. Chromatograms were run as follows: Silica Gel 60 thin-layer plates (10 by 10 cm) were spotted with 10 μL of a whole-cell lipid extract. Chloroform/methanol/water (65:25:4, by vol.) was used in the first direction, and chloroform/acetic acid/methanol/water (80:18:12:5, by vol.) was used in the second direction. A, The plate was sprayed with molybdatophosphoric acid; B, The plate was sprayed with ninhydrin reagent; C, The plate was sprayed with molybdenum blue reagent; DPG, diphosphatidylglycerol; PG, phosphatidylglycerol; PE, phosphatidylethanolamine; PL, unidentified phospholipid. F, first dimension of thin layer chromatogram; S, second dimension of thin layer chromatogram.

Description of *Kordiimonas coralliicola* sp. nov.

Kordiimonas coralliicola (co.ral.li.i'co.la L. neut. n.*corallium*, coral; L. masc./fem. suff.-*cola*, inhabitant; from L. masc./fem. n.*incola*, dweller; N.L. masc./fem. n.*coralliicola*, inhabitant of corals).

The ANI and dDDH value between the genome of *Kordiimonas lipolytica* and strain SCSIO 12603^{T} is 68.87% and 19.7%, respectively; between *Kordiimonas lacus* and strain SCSIO 12603^{T} is 68.81% and 21.1%, respectively; between *Kordiimonas gwangyangensis* and strain SCSIO 12603^{T} is 68.60% and 19.1%, respectively; and between strains SCSIO 12603^{T} and SCSIO 12610^{T} is 68.41% and 21.9%, respectively. These values clearly confirm the separate species status of strain SCSIO 12603^{T} (Fig. 10).

Cells are Gram-stain-negative, aerobic, rod or slight curved rod, motile by means of a single polar flagellum (Fig. 11). Produce black soluble pigment. Growth is observed at 3% (w/v) NaCl. The pH and temperature ranges for growth are pH 5–8 and 10–33 °C (optimum growth at pH 6-7 and 25 °C). Positive for oxidase and catalase activities, hydrolysis of starch, and nitrate reduction. Negative for urease activity and hydrolysis of gelatin. Positive for alkaline phosphatase, esterase (C4), esterase lipase (C8), lipase (C14), leucine arylamidase, valine arylamidase, cystine arylamidase, trypsin, α-chymotrypsin, acid phosphatase, and naphthol-AS-BI-phosphohydrotase, but negative for α-galactosidase, β-galactosidase, α-glucosidase, β-glucosidase, N-acetyl-β-glucosaminidase, α-mannosidase, β-fucosidase, and β-glucuronidase. Dextrin, D-maltose, D-cellobiose, gentiobiose, sucrose, D-turanose, stachyose, D-raffinose, D-melibiose, D-salicin, N-acetyl-D-galactosamine, α-D-glucose, D-mannose, D-fructose, D-galactose, D-fucose, L-fucose, L-rhamnose, inosine, D-mannitol, D-arabitol, myo-inositol, glycerol, D-aspartic acid, L-histidine, D-glucuronic acid, glucuronamide, quinic acid, D-saccharic acid, L-lactic acid, citric

acid, D-malic acid, acetoacetic acid, propionic acid, and acetic acid are utilized as sole carbon and energy sources but D-trehalose, *α*-D-lactose, 3-methyl glucose, D-sorbitol, glycyl-L-prolin, L-arginine, L-pyroglutamic acid, L-galactonic acid lactone, *α*-keto-glutaric acid, L-malic acid, tween 40, N-acetyl-D-glucosamine, D-fructose-6-PO_4, D-serine, gelatin, L-aspartic acid, L-serine, mucic acid, *p*-hydroxy-phenylacetic acid, D-lactic acid methyl ester, bromo-succinic acid, *γ*-amino-butryric acid, *α*-hydroxy-butyric acid, *β*-hydroxy-D,L butyric acid, *β*-methyl-D-glucoside, methyl pyruvate, *α*-keto-butyric acid, L-alanine, L-glutamic acid, D-gluconic acid, pectin, D-galacturonic acid, D-glucose-6-PO_4, N-acetyl-β-D-mannosamine, N-acetyl neuraminic acid, and formic acid are not. Inhibited by 1 % sodium lactate, fusidic acid, guanidine HCl, and lithium chloride, but not by niaproof 4, minocycline, troleandomycin, rifamycin SV, lincomycin, tetrazolium violet, tetrazolium blue, nalidixic acid, aztreonam, potassium tellurite, vancomycin, sodium bromate, and sodium butyrate. The ubiquinone system is ubiquinone Q-10 with small amounts of Q-9. The main cellular fatty acids are summed feature 9 (iso-$C_{17:1}\omega 9c$/$C_{16:0}$ 10-methyl), iso-$C_{15:0}$, summed feature 3 ($C_{16:1}\omega 7c$/$C_{16:1}\omega 6c$), iso-$C_{17:0}$, and $C_{17:1}\omega 8c$. The polar lipids present are phosphatidylglycerol, phosphatidylethanolamine, two unidentified phospholipids, and three unidentified aminolipids (Fig. 12). The G+C content of genomic DNA is 46.02%. The type strain is SCSIO 12603^T.

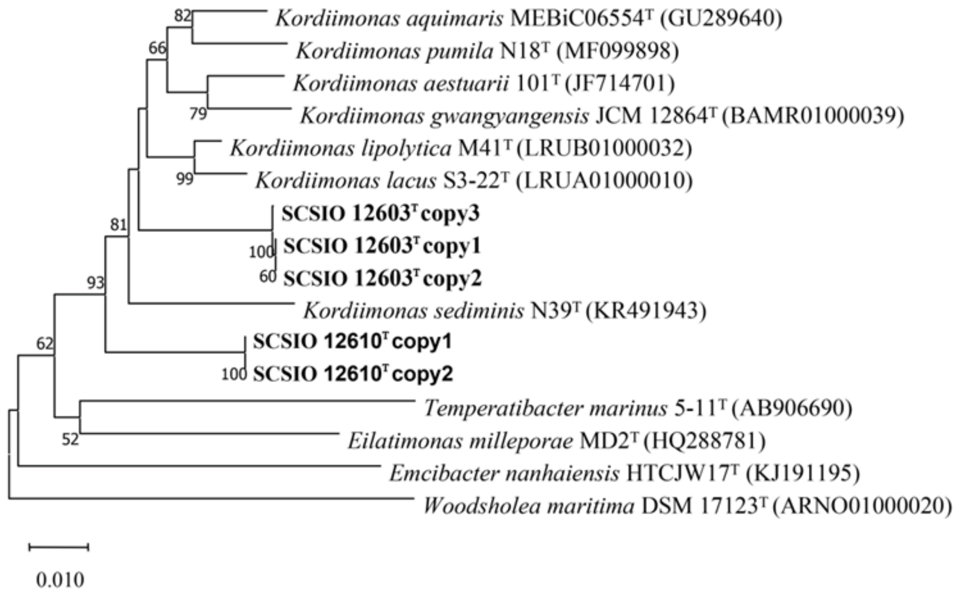

Figure 10. Neighbour-joining phylogenetic tree reconstructed based on 16S rRNA gene sequences, showing the position of strains SCSIO 12610^T, SCSIO 12603^T and other closely related species. Three 16S rRNA gene copies of strain SCSIO 12603^T and two 16S rRNA gene copies of strain SCSIO 12610^T were included in phylogenetic analysis. Only bootstrap values (percentages of 1000 replications) >50% were shown. The strain characterized in this study is shown in bold type. All ambiguous positions were removed for each sequence pair (pairwise deletion option). There were a total of 1511 positions in the final dataset. Bar, 0.01 changes per nucleotide position.

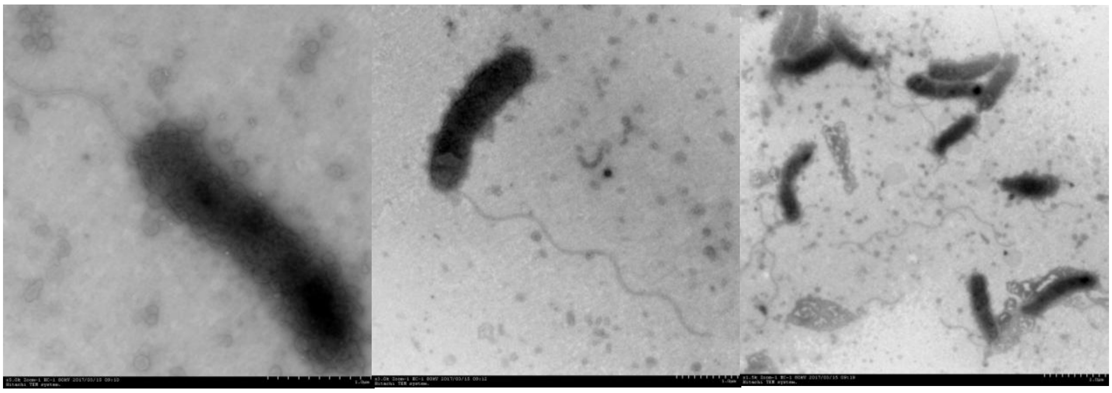

Figure 11. Transmission electron microscope photographs of strain SCSIO 12603^T.

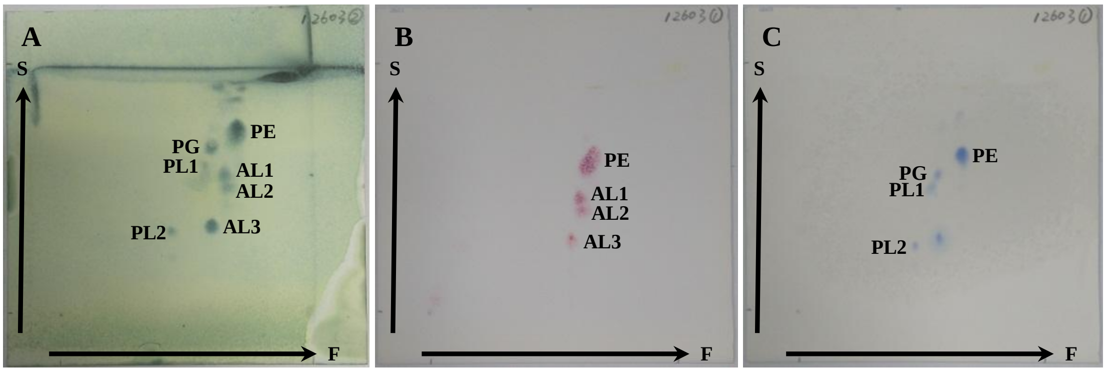

Figure 12. Two-dimensional thin layer chromatogram of polar lipids of strain SCSIO 12603^{T}. Chromatograms were run as follows: Silica Gel 60 thin-layer plates (10 by 10 cm) were spotted with 10 µL of a whole-cell lipid extract. Chloroform/methanol/water (65:25:4, by vol.) was used in the first direction, and chloroform/acetic acid/methanol/water (80:18:12:5, by vol.) was used in the second direction. A, The plate was sprayed with molybdatophosphoric acid; B, The plate was sprayed with ninhydrin reagent; C, The plate was sprayed with molybdenum blue reagent; PG, phosphatidylglycerol; PE, phosphatidylethanolamine; PL, unknown phospholipid; AL, unidentified aminolipid. F, first dimension of thin layer chromatogram; S, second dimension of thin layer chromatogram.

Description of *Kordiimonas coralli* sp. nov.

Kordiimonas *coralli* (co.ral'li L. gen. neut. n.*coralli*, of coral, from which the organism was isolated).

The ANI and dDDH value between the genome of *Kordiimonas lipolytica* and strain SCSIO 12610^{T} is 67.67% and 22.9%, respectively; between *Kordiimonas lacus* and strain SCSIO 12610^{T} is 67.60% and 23.0%, respectively; between *Kordiimonas gwangyangensis* and strain SCSIO 12610^{T} is 67.6% and 20.1%, respectively. These values clearly confirm the separate species status of strain SCSIO 12610^{T} (Fig. 10).

Gram-stain-negative, facultative anaerobic, rod-shaped, motile by means of a polar single flagellum (Fig. 13). Oxidase and catalase are negative. Requiring seawater for growth. Growth is observed at 3–5% (w/v) NaCl with optimum growth at 3%. The pH and temperature ranges for growth are pH 6–7 and 15–37 °C (optimum growth at

25 °C). Positive for hydrolysis of starch and nitrate reduction. Negative for urease activity and hydrolysis of gelatin. Positive for alkaline phosphatase, esterase (C4), esterase lipase (C8), leucine arylamidase, valine arylamidase, cystine arylamidase, trypsin, α-chymotrypsin, acid phosphatase, and naphthol-AS-BI-phosphohydrotase, but negative for lipase (C14), α-galactosidase, β-galactosidase, α-glucosidase, β-glucosidase, N-acetyl-β-glucosaminidase, α-mannosidase, β-fucosidase, and β-glucuronidase. Dextrin, D-maltose, D-trehalose, D-cellobiose, gentiobiose, sucrose, stachyose, D-raffinose, α-D-lactose, D-melibiose, β-methyl-D-glucoside, D-salicin, N-acetyl-D-galactosamine, N-acetyl-D-glucosamine, N-acetyl-β-D-mannosamine, D-mannose, D-galactose, 3-methyl glucose, D-fucose, L-fucose, L-rhamnose, inosine, D-sorbitol, D-mannitol, D-arabitol, myo-inositol, glycerol, D-fructose-6-PO_4, L-histidine, pectin, D-galacturonic acid, L-galactonic acid lactone, glucuronamide, D-saccharic acid, citric acid, acetoacetic acid, propionic acid, and acetic acid are utilized as sole carbon and energy sources but D-turanose, α-D-glucose, D-fructose, D-aspartic acid, glycyl-L-prolin, L-arginine, L-pyroglutamic acid, α-keto-glutaric acid, L-malic acid, tween 40, D-serine, gelatin, L-aspartic acid, D-malic acid, L-serine, mucic acid, *p*-hydroxy-phenylacetic acid, D-lactic acid methyl ester, quinic acid, bromo-succinic acid, γ-amino-butryric acid, L-lactic acid, α-hydroxy-butyric acid, β-hydroxy-D,L butyric acid, methyl pyruvate, α-keto-butyric acid, L-alanine, D-glucuronic acid, L-glutamic acid, D-gluconic acid, D-glucose-6-PO_4, N-acetyl neuraminic acid, and formic acid are not. Inhibited by troleandomycin, guanidine HCl, and lithium chloride, but not by minocycline, rifamycin SV, 1% sodium lactate, fusidic

acid, lincomycin, tetrazolium violet, tetrazolium blue, niaproof 4, nalidixic acid, aztreonam, potassium tellurite, vancomycin, sodium bromate, and sodium butyrate. The respiratory quinone is Q-10. The main cellular fatty acids are $C_{17:1}$ *ω8c*, summed feature 3 ($C_{16:1}$*ω7c*/$C_{16:1}$*ω6c*), $C_{17:1}$ *ω6c*, summed feature 9 (iso-$C_{17:1}$ *ω9c*/$C_{16:0}$ 10-methyl), and iso-$C_{15:0}$. The polar lipids present are diphosphatidylglycerol, phosphatidylglycerol, phosphatidylethanolamine, and two unidentified aminolipids (Fig. 14). The G+C content of genomic DNA of the type strain is 45.72%. The type strain is SCSIO 12610^T.

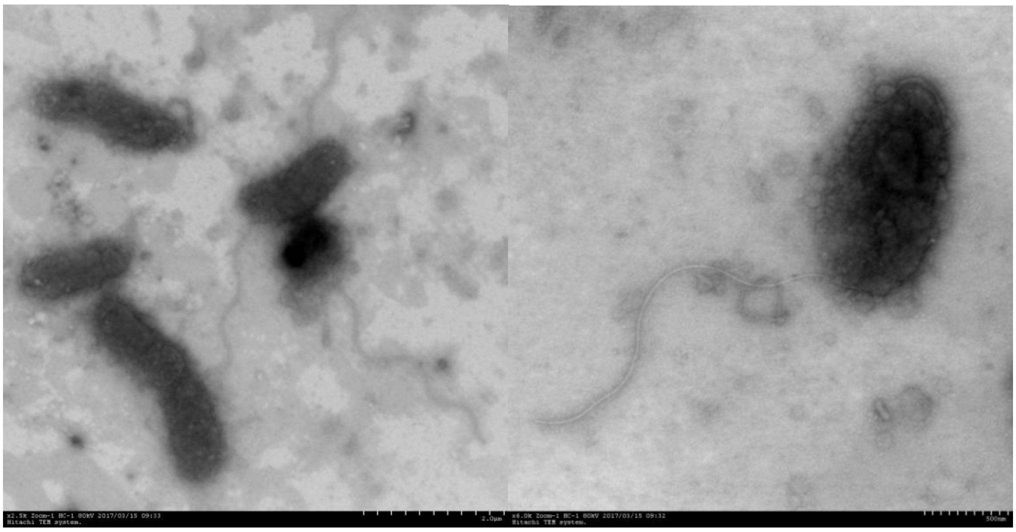

Figure 13. Transmission electron microscope photographs of strain SCSIO 12610^T.

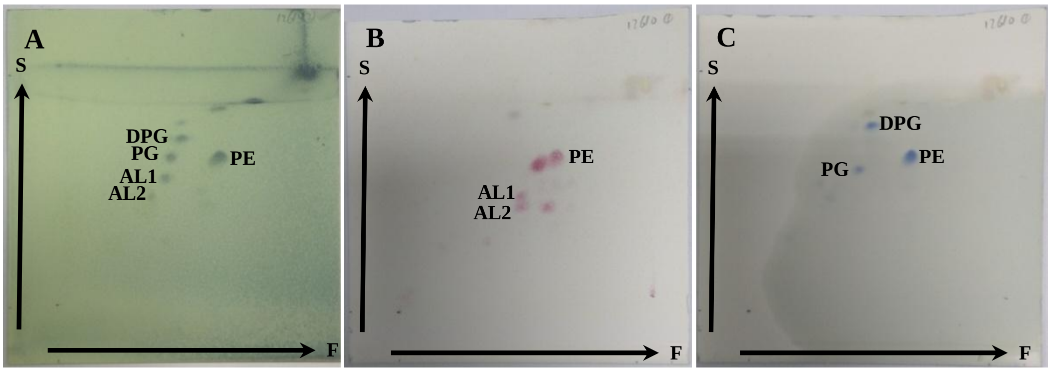

Figure 14. Two-dimensional thin layer chromatogram of polar lipids of strain SCSIO 12610^T. Chromatograms were run as follows: Silica Gel 60 thin-layer plates (10 by 10 cm) were spotted with 10 μL of a whole-cell lipid extract.

Chloroform/methanol/water (65:25:4, by vol.) was used in the first direction, and chloroform/acetic acid/methanol/water (80:18:12:5, by vol.) was used in the second direction. A, The plate was sprayed with molybdatophosphoric acid; B, The plate was sprayed with ninhydrin reagent; C, The plate was sprayed with molybdenum blue reagent; DPG, diphosphatidylglycerol; PG, phosphatidylglycerol; PE, phosphatidylethanolamine; AL, unidentified aminolipid. F, first dimension of thin layer chromatogram; S, second dimension of thin layer chromatogram.

Description of *Marinivarius* gen. nov.

Marinivarius (Ma.ri.ni.va'ri.us L. masc. adj. *marinus*, of the sea; L. masc. adj. *varius*, variable; N.L. masc. n. *Marinivarius*, variably shaped bacterium belonging to or living in the sea).

Phylogenetically, the genus is affiliated to the family Endozoicomonadaceae, and forms a phyletic lineage that is distinct from the known genera within Endozoicomonadaceae (Fig. 15). The POCP value between the genome of strain SCSIO 12664^T and *Endozoicomonas montiporae* and *Parendozoicomonas haliclonae* is 40.85% and 34.40%, respectively, which confirms separate genus status of strain SCSIO 12664^T.

Gram-stain-negative, aerobic, non-motile, oxidase-positive, and catalase-negative. Cells are curved rod or irregular shaped (Fig. 16). Requiring seawater for growth. The main cellular fatty acids are summed feature 8 ($C_{18:1}\omega7c$/$C_{18:1}\omega6c$), summed feature 3 ($C_{16:1}\omega7c$/$C_{16:1}\omega6c$), $C_{14:0}$, $C_{16:0}$, and summed feature 2 ($C_{14:0}$ 3-OH/iso-$C_{16:0}$ I). The ubiquinone system is ubiquinone Q-9 with small amounts of Q-10 and Q-8. The polar lipids present are phosphatidylethanlamine, phosphatidylglycerol, diphosphatidylglycerol, one unidentified phospholipid, and one unidentified aminolipid (Fig. 17). The type species is *Marinivarius coralli*.

Description of *Marinivarius coralli* sp. nov.

Marinivarius coralli (co.ral'li. L. gen. neut. n. *coralli*, of coral, from which the organism was isolated).

In addition to the properties given in the genus description, colonies are circular, creamy yellow and with regular shape, clear edges, 0.5 mm in diameter on marine agar after cultivation for 2 days at 25 °C. Produce black soluble pigment. Growth occurs at 20–35 °C (optimum, 25 °C), at pH 4–8 (optimum, pH 8) and with 3–3.7% (w/v) NaCl concentrations. Positive for hydrolysis of gelatin and nitrate reduction. Negative for urease activity and hydrolysis of starch. Positive for alkaline phosphatase, esterase (C4), esterase lipase (C8), lipase (C14), leucine arylamidase, valine arylamidase, cystine arylamidase, trypsin, *α*-chymotrypsin, acid phosphatase, *α*-galactosidase, *β*-galactosidase, N-acetyl-*β*-glucosaminidase, and naphthol-AS-BI-phosphohydrotase, but negative for *α*-glucosidase, *β*-glucosidase, *α*-mannosidase, *β*-fucosidase, and *β*-glucuronidase. Dextrin, D-maltose, D-trehalose, D-cellobiose, gentiobiose, sucrose, stachyose, D-raffinose, *α*-D-lactose, D-melibiose, *β*-methyl-D-glucoside, D-salicin, N-acetyl-*β*-D-mannosamine, N-acetyl-D-glucosamine, N-acetyl-D-galactosamine, *α*-D-glucose, D-mannose, D-fructose, D-galactose, 3-methyl glucose, D-fucose, L-fucose, L-rhamnose, inosine, D-sorbitol, D-mannitol, D-arabitol, myo-inositol, D-fructose-6-PO_4, D-aspartic acid, L-glutamic acid, L-pyroglutamic acid, pectin, D-galacturonic acid, D-saccharic acid, citric acid, *α*-keto-butyric acid, *γ*-amino-butryric acid, acetoacetic acid, propionic acid, *α*-keto-glutaric acid, formic acid, and acetic acid are utilized as sole carbon and

energy sources but D-turanose, N-acetyl neuraminic acid, D-glucose-6-PO_4, L-arginine, L-aspartic acid, glycerol, gelatin, L-serine, mucic acid, quinic acid, D-serine, L-alanine, glycyl-L-prolin, methyl pyruvate, L-histidine, D-gluconic acid, glucuronamide, L-galactonic acid lactone, D-glucuronic acid, D-malic acid, *p*-hydroxy-phenylacetic acid, D-lactic acid methyl ester, *α*-hydroxy-butyric acid, L-lactic acid, bromo-succinic acid, tween 40, *β*-hydroxy-D,L butyric acid, and L-malic acid are not. Inhibited by 1% sodium lactate, fusidic acid, rifamycin SV, lincomycin, guanidine HCl, niaproof 4, nalidixic acid, lithium chloride, sodium bromate, and sodium butyrate but not by troleandomycin, minocycline, tetrazolium violet, tetrazolium blue, potassium tellurite, aztreonam, and vancomycin. The G+C content of chromosome DNA is 46.77%. The type strain has one plasmid with 41.65% G+C content. The type strain is SCSIO 12664^T.

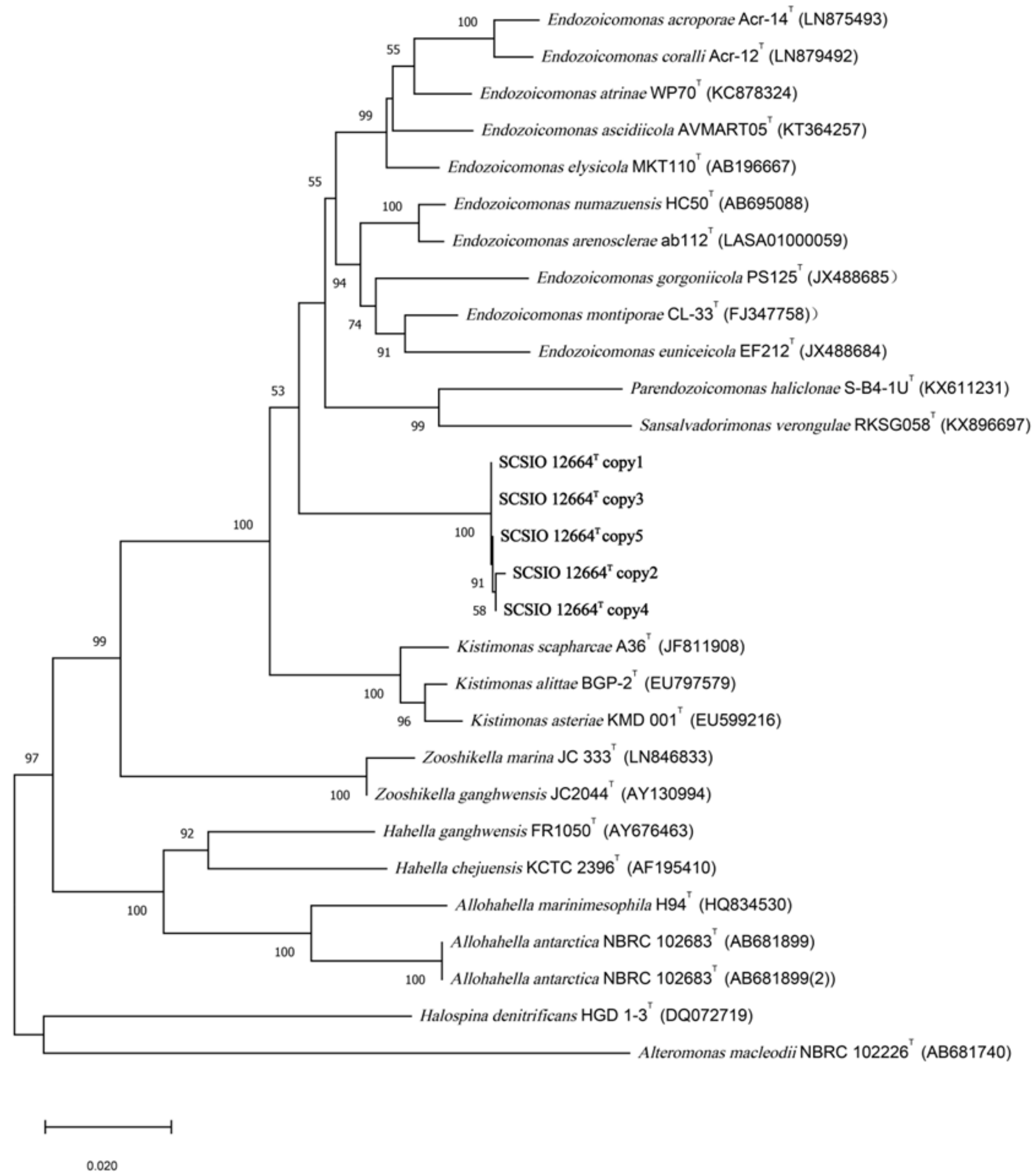

Figure 15. Neighbour-joining phylogenetic tree reconstructed based on 16S rRNA gene sequences, showing the position of strain SCSIO 12664^T and other closely related species. Five 16S rRNA genecopies ofstrain SCSIO 12664^T were included in phylogenetic analysis. Only bootstrap values (percentages of 1000 replications) >50% were shown. The strain characterized in this study is shown in bold type. All ambiguous positions were removed for each sequence pair (pairwise deletion option). There were a total of 1578 positions in the final dataset. Bar, 0.02 changes per nucleotide position.

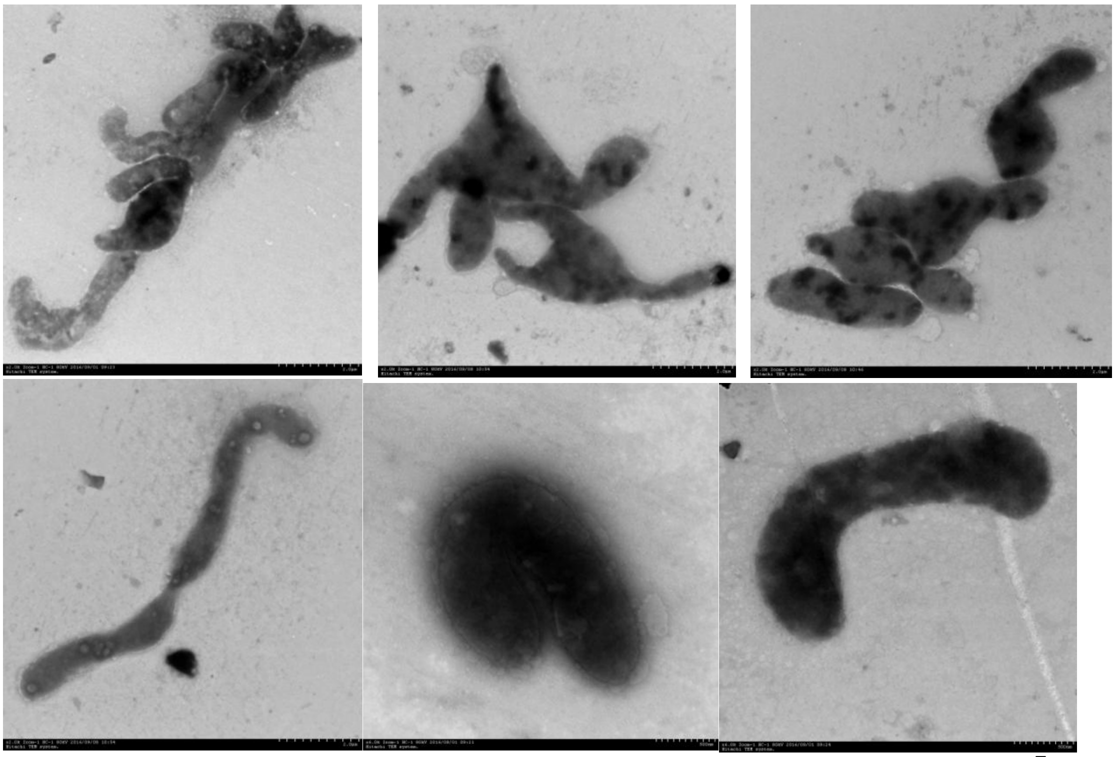

Figure 16. Transmission electron microscope photographs of strain SCSIO 12664^T.

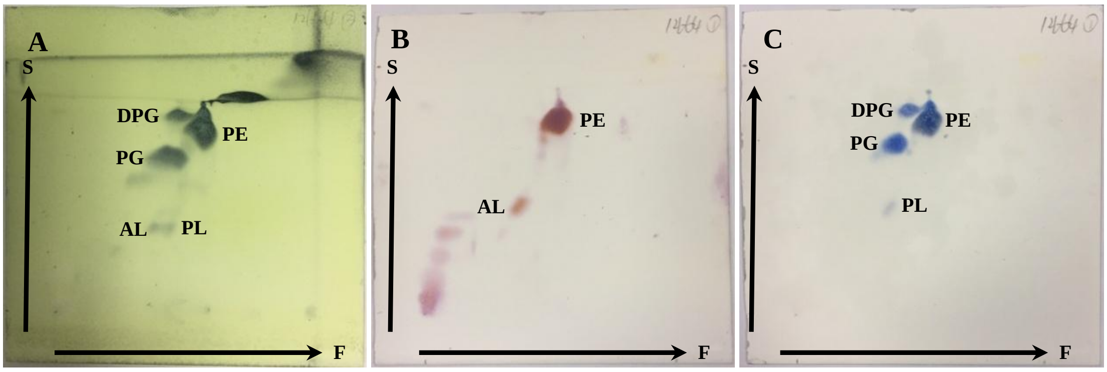

Figure 17. Two-dimensional thin layer chromatogram of polar lipids of strain SCSIO 12664^T. Chromatograms were run as follows: Silica Gel 60 thin-layer plates (10 by 10 cm) were spotted with 10 μL of a whole-cell lipid extract. Chloroform/methanol/water (65:25:4, by vol.) was used in the first direction, and chloroform/acetic acid/methanol/water (80:18:12:5, by vol.) was used in the second direction. A, The plate was sprayed with molybdatophosphoric acid; B, The plate was sprayed with ninhydrin reagent; C, The plate was sprayed with molybdenum blue reagent; DPG, diphosphatidylglycerol; PG, phosphatidylglycerol; PE, phosphatidylethanolamine; PL, unknown phospholipid; AL, unidentified aminolipid. F, first dimension of thin layer chromatogram; S, second dimension of thin layer chromatogram.

Description of *Pocilloporibacter* gen. nov.

Pocilloporibacter (po.cil.lo.po.ri.bac'ter N.L. gen. n. *pocillopori*, pertaining to the coral *Pocillopora damicornis*; N.L. masc. n. *bacter*, a rod; N.L. masc. n. *Pocilloporibacter*, a rod isolated from the coral *Pocillopora damicornis*).

Strain SCSIO 12844^T showed the highest 16S rRNA gene similarity (88.47%) to *Fangia hongkongensis*. According to the 16S rRNA gene-based phylogeny, strain SCSIO 12844^T is placed at a distinct clade from type species within type genera of families in Gammaproteobacteria class, and clustered with *Fastidiosibacter lacustris* (Fastidiosibacteraceae) and *Fangia hongkongensis* (not assigned to family) (Fig. 18). The POCP value between the genome of strain SCSIO 12844^T and *Fastidiosibacter lacustris* and *Fangia hongkongensis* is 41.26% and 39.36%, respectively. Thus, we propse it represents a novel genus.

Gram-stain-negative, aerobic, non-motile, rod-shaped (Fig. 19). Catalase-positive, oxidase-negative. The respiratory quinone is ubiquinone Q-8. The main cellular fatty acids are iso-$C_{17:0}$, iso-$C_{17:0}$ 3-OH, $C_{18:1}$ $\omega 9c$, iso-$C_{13:0}$, and $C_{18:0}$ 10-methyl (TBSA). The major polar lipids are phosphatidylglycerol, phosphatidylethanolamine, diphosphatidylglycerol, four unknown phospholipids, and an unidentified aminolipid (Fig. 20). The G+C content of genomic DNA of the type strain is 32.92%. Type species is *Pocilloporibacter coralii*.

Description of *Pocilloporibacter coralii* sp. nov.

Pocilloporibacter coralii (co.ral'li L. gen. neut. n. *coralli*, of coral).

In addition to the properties given in the genus description, colonies on marine agar

are transparent, colorless and circular with regular margins. The colony size is approximately 0.5 mm in diameter after 3 days at 25 °C. Negative for hydrolysis of gelatin and starch, nitrate reduction, and urease activity. Growth occurs at 15–37 °C (optimum, 30 °C), at pH 5–7 and with 0.5–5% (w/v) NaCl concentrations (optimum, 1-3%). Positive for alkaline phosphatase, esterase (C4), esterase lipase (C8), acid phosphatase, leucine arylamidase, and naphthol-AS-BI-phosphohydrotase, but negative for *β*-fucosidase, N-acetyl-*β*-glucosaminidase, *α*-galactosidase, *β*-galactosidase, α-glucosidase, *β*-glucosidase, valine arylamidase, cystine arylamidase, trypsin, *α*-chymotrypsin, *α*-mannosidase, and *β*-glucuronidase. Dextrin, D-trehalose, D-cellobiose, gentiobiose, sucrose, D-turanose, stachyose, D-raffinose, *α*-D-lactose, D-melibiose, *β*-methyl-D-glucoside, N-acetyl-D-glucosamine, N-acetyl-*β*-D-mannosamine, N-acetyl-D-galactosamine, *α*-D-glucose, D-fructose, D-fucose, L-rhamnose, inosine, D-sorbitol, D-mannitol, *α*-keto-glutaric acid, D-arabitol, myo-inositol, glycerol, D-glucose-6-PO_4, glycyl-L-prolin, L-histidine, pectin, glucuronamide, D-saccharic acid, D-malic acid, acetoacetic acid, propionic acid, and acetic acid are utilized as sole carbon and energy sources but D-maltose, D-salicin, D-fructose-6-PO_4, L-aspartic acid, L-malic acid, citric acid, quinic acid, D-galacturonic acid, D-glucuronic acid, L-fucose, D-mannose, D-galactose, 3-methyl glucose, N-acetyl neuraminic acid, L-arginine, mucic acid, L-galactonic acid lactone, gelatin, L-serine, D-aspartic acid, L-glutamic acid, L-pyroglutamic acid, D-serine, L-alanine, methyl pyruvate, *α*-keto-butyric acid, D-gluconic acid, L-galactonic acid lactone, *p*-hydroxy-phenylacetic acid, D-lactic acid methyl ester, *α*-hydroxy-butyric acid,

L-lactic acid, bromo-succinic acid, formic acid, tween 40, γ-amino-butryric acid, and β-hydroxy-D,L butyric acid are not. Inhibited by fusidic acid, guanidine HCl, niaproof 4, nalidixic acid, potassium tellurite, sodium butyrate, sodium bromate, and lithium chloride but not by 1% sodium lactate, minocycline, troleandomycin, rifamycin SV, lincomycin, tetrazolium violet, tetrazolium blue, vancomycin, and aztreonam. The type strain is SCSIO 12844^T.

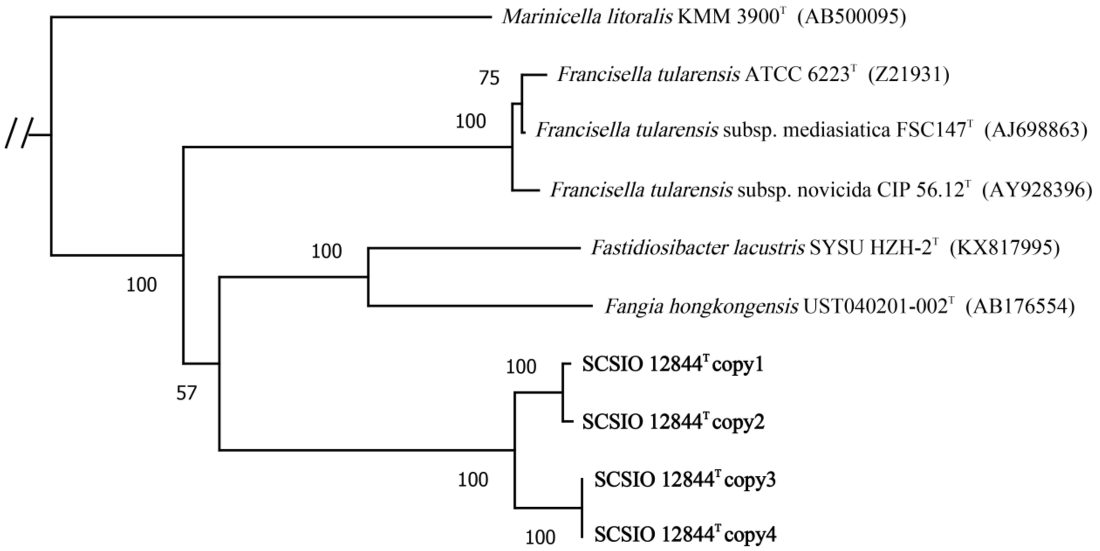

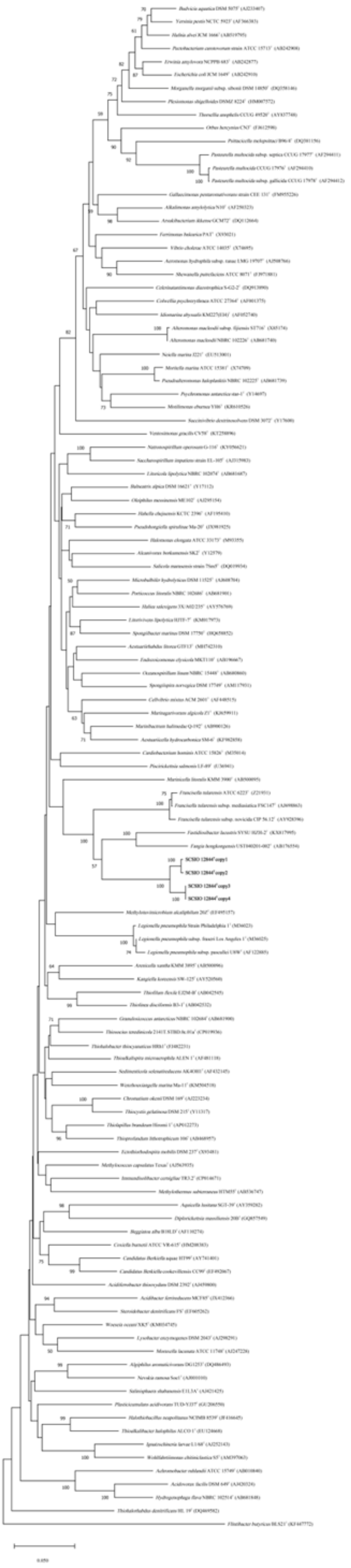

Figure 18. Neighbour-joining phylogenetic tree reconstructed based on 16S rRNA gene sequences, showing the position of strain SCSIO 12844^T and other type species within type genera of families in Gammaproteobacteria class. Four 16S rRNA gene copies of strain SCSIO 12844^T were included in phylogenetic analysis. Only bootstrap values (percentages of 1000 replications) >50% were shown. The strain characterized in this study is shown in bold type. All ambiguous positions were removed for each sequence pair (pairwise deletion option). There were a total of 1720 positions in the final dataset. Bar, 0.05 changes per nucleotide position.

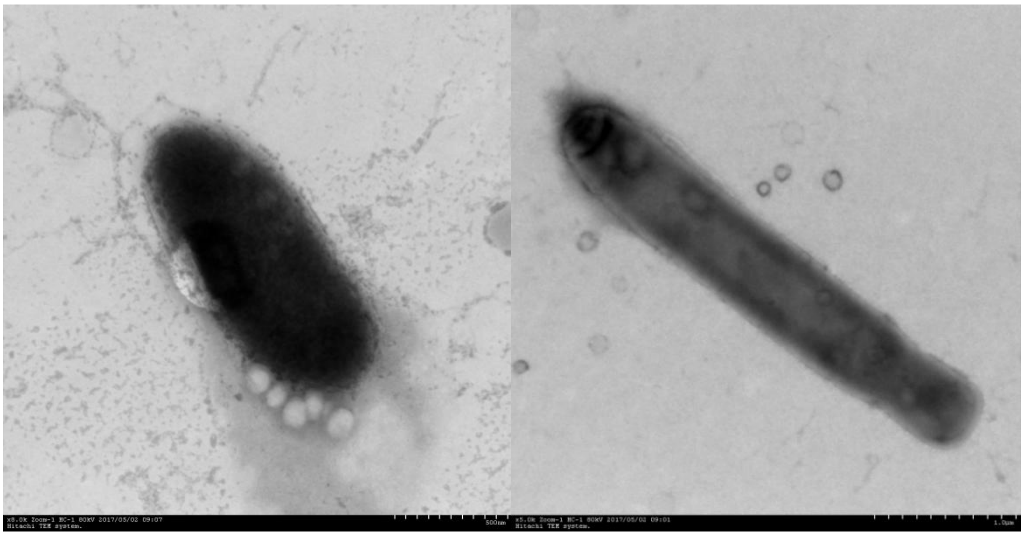

Figure 19. Transmission electron microscope photographs of strain SCSIO 12844^T.

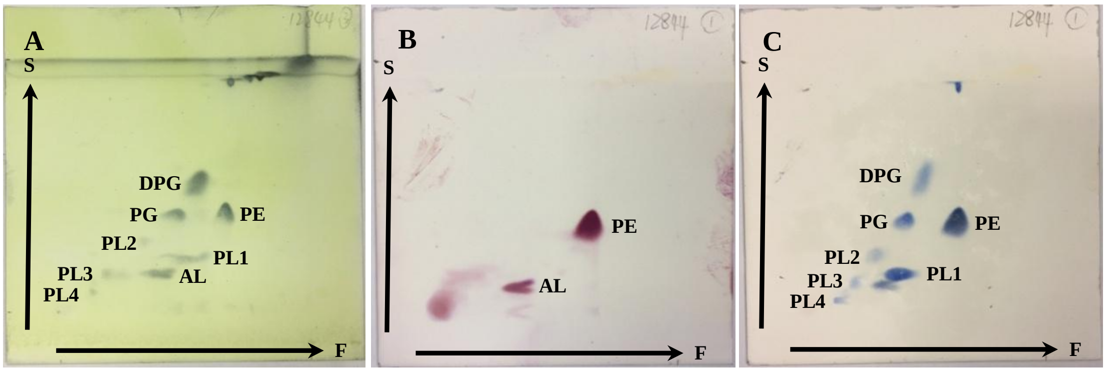

Figure 20. Two-dimensional thin layer chromatogram of polar lipids of strain SCSIO 12844^T. Chromatograms were run as follows: Silica Gel 60 thin-layer plates (10 by 10 cm) were spotted with 10 μL of a whole-cell lipid extract. Chloroform/methanol/water (65:25:4, by vol.) was used in the first direction, and chloroform/acetic acid/methanol/water (80:18:12:5, by vol.) was used in the second direction. A, The plate was sprayed with molybdatophosphoric acid; B, The plate was sprayed with ninhydrin reagent; C, The plate was sprayed with molybdenum blue reagent; DPG, diphosphatidylglycerol; PG, phosphatidylglycerol; PE, phosphatidylethanolamine; PL, unknown phospholipid; AL, unidentified aminolipid. F, first dimension of thin layer chromatogram; S, second dimension of thin layer chromatogram.

Description of *Corallitalea* gen. nov.

Corallitalea (Co.ral.li.ta’le.a L. neut. n. *corallium*, coral; L. fem. n. *talea*, a rod; N.L. fem. n. *Corallitalea*, a rod from a coral, referring to the isolation of the first strain from the coral *Pocillopora damicornis*).

Strain SCSIO 12643^{T} showed the highest 16S rRNA gene similarity (88.26%) to *Vicingus serpentipes*. According to the 16S rRNA gene-based phylogeny, the strain is placed at a distinct clade from type species within families in the order Flavobacteriales, and clustered with SCSIO 12741^{T}, *Owenweeksia hongkongensis* (Schleiferiaceae), and *Salibacter halophilus* (Cryomorphaceae) (Fig. 21). The POCP value between the genome of strain SCSIO 12643^{T} and *Owenweeksia hongkongensis*, *Salibacter halophilus*, *Vicingus serpentipes*, and SCSIO 12741^{T} is 32.75%, 41.45%, 36.55% and 36.68%, respectively. Thus, we propse it represents a novel genus.

Gram-stain-negative, aerobic, rod-shaped, motile by means of a lateral flagellum (Fig. 22). Oxidase-positive, catalase-negative. Requiring seawater for growth. The major respiratory quinoneis MK-6. The main cellular fatty acids are iso-$C_{15:0}$, summed feature 4 (iso-$C_{17:1}$ I/anteiso-B), summed feature 3 ($C_{16:1}\omega 7c$/$C_{16:1}\omega 6c$), summed feature 1 (iso-$C_{15:1}$ H/$C_{13:0}$ 3-OH), and iso-$C_{17:0}$ 3-OH. The polar lipids present are phosphatidylethanolamine, an unidentified aminolipid, and three unknown polar lipids (Fig. 23). The G+C content of the genomic DNA of the type strain is 36.37%. The type species is *Corallitalea lutea*.

Description of *Corallitalea lutea* sp. nov.

Corallitalea lutea (lu’te.a L. fem. adj. *lutea*, yellow, referring to its yellow colour).

Exhibits the following properties in addition to those given in the genus description. Cells are 0.5–0.7 µm in width and 1.4–2.3 µm in length. Colonies are circular, yellow with regular shape, clear edges, 1–2 mm in diameter on marine agar after cultivation for 3 days at 25 °C. Growth occurs at 15–35 °C (optimum, 30–35 °C), at pH 6–8 (optimum, pH 7.0) and with 3–5% (w/v) NaCl concentrations (optimum, 3%). Negative for hydrolysis of gelatin and starch, nitrate reduction, and urease activity. Positive for alkaline phosphatase, esterase (C4), esterase lipase (C8), leucine arylamidase, valine arylamidase, cystine arylamidase, trypsin, acid phosphatase, naphthol-AS-BI-phosphohydrotase, and N-acetyl-*β*-glucosaminidase, but negative for lipase (C14), *α*-chymotrypsin, *α*-galactosidase, *β*-galactosidase, *α*-glucosidase, *β*-glucosidase, *α*-mannosidase, *β*-fucosidase, and *β*-glucuronidase. Dextrin, D-trehalose, D-cellobiose, gentiobiose, sucrose, D-turanose, stachyose, D-raffinose, *α*-D-lactose, D-melibiose, *β*-methyl-D-glucoside, D-salicin, N-acetyl-D-glucosamine, N-acetyl-D-galactosamine, *α*-D-glucose, D-mannose, D-fructose, D-galactose, 3-methyl glucose, D-fucose, L-fucose, L-rhamnose, inosine, D-sorbitol, D-arabitol, glycerol, L-pyroglutamic acid, pectin, L-galactonic acid lactone, glucuronamide, D-saccharic acid, citric acid, *α*-keto-glutaric acid, L-malic acid, acetoacetic acid, propionic acid, formic acid and acetic acid are utilized as sole carbon and energy sources but D-maltose, N-acetyl-*β*-D-mannosamine, N-acetyl neuraminic acid, D-serine, D-aspartic acid, gelatin, glycyl-L-prolin, L-arginine, L-aspartic acid, L-histidine, L-serine, mucic acid, quinic acid, D-mannitol, myo-inositol, D-glucose-6-PO_4, D-fructose-6-PO_4, L-alanine, L-glutamic acid, D-malic acid, methyl

pyruvate, D-gluconic acid, D-glucuronic acid, *α*-keto-butyric acid, D-galacturonic acid, *p*-hydroxy-phenylacetic acid, D-lactic acid methyl ester, L-lactic acid, bromo-succinic acid, tween 40, *γ*-amino-butryric acid, *α*-hydroxy-butyric acid, and *β*-hydroxy-D,L butyric acid are not. Inhibited by fusidic acid, troleandomycin, rifamycin SV, lincomycin, guanidine HCl, vancomycin, nalidixic acid, and lithium chloride but not by 1% sodium lactate, minocycline, niaproof 4, tetrazolium violet, tetrazolium blue, potassium tellurite, sodium bromate, aztreonam, and sodium butyrate. The type strain is SCSIO 12643^{T}.

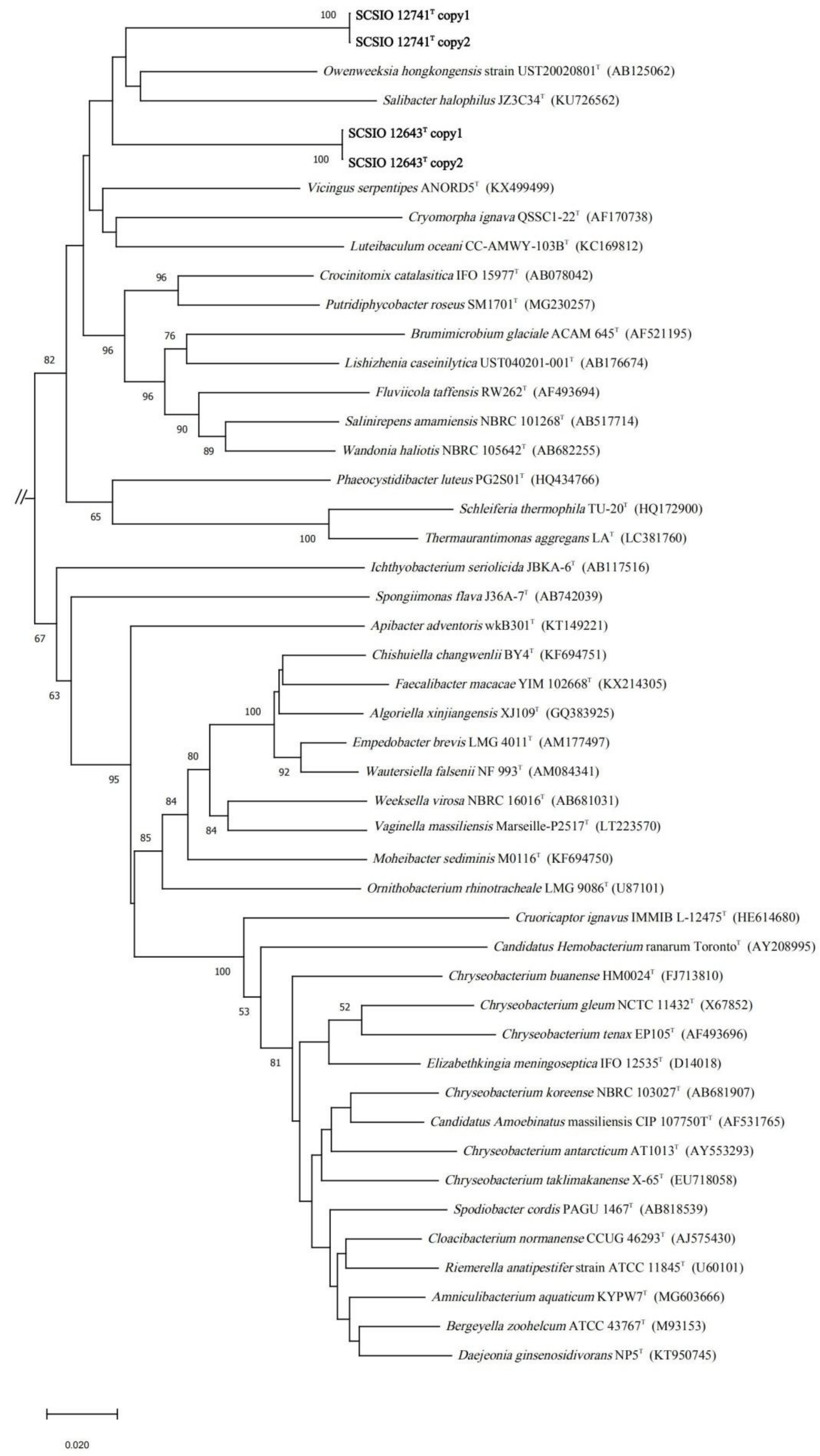
SCSIO 12741^{T} copy1
SCSIO 12741^{T} copy2
Owenweeksia hongkongensis strain UST20020801^{T} (AB125062)
Salibacter halophilus JZ3C34^{T} (KU726562)
SCSIO 12643^{T} copy1
SCSIO 12643^{T} copy2
Vicingus serpentipes ANORD5^{T} (KX499499)
Cryomorpha ignava QSSC1-22^{T} (AF170738)
Luteibaculum oceani CC-AMWY-103B^{T} (KC169812)
Crocinitomix catalasitica IFO 15977^{T} (AB078042)
Putridiphycobacter roseus SM1701^{T} (MG230257)
Brumimicrobium glaciale ACAM 645^{T} (AF521195)
Lishizhenia caseinilytica UST040201-001^{T} (AB176674)
Fluviicola taffensis RW262^{T} (AF493694)
Salinirepens amamiensis NBRC 101268^{T} (AB517714)
Wandonia haliotis NBRC 105642^{T} (AB682255)
Phaeocystidibacter luteus PG2S01^{T} (HQ434766)
Schleiferia thermophila TU-20^{T} (HQ172900)
Thermaurantimonas aggregans LA^{T} (LC381760)
Ichthyobacterium seriolicida JBKA-6^{T} (AB117516)
Spongiimonas flava J36A-7^{T} (AB742039)
Apibacter adventoris wkB301^{T} (KT149221)
Chishuiella changwenlii BY4^{T} (KF694751)
Faecalibacter macacae YIM 102668^{T} (KX214305)
Algoriella xinjiangensis XJ109^{T} (GQ383925)
Empedobacter brevis LMG 4011^{T} (AM177497)
Wautersiella falsenii NF 993^{T} (AM084341)
Weeksella virosa NBRC 16016^{T} (AB681031)
Vaginella massiliensis Marseille-P2517^{T} (LT223570)
Moheibacter sediminis M0116^{T} (KF694750)
Ornithobacterium rhinotracheale LMG 9086^{T} (U87101)
Cruoricaptor ignavus IMMIB L-12475^{T} (HE614680)
Candidatus Hemobacterium ranarum Toronto^{T} (AY208995)
Chryseobacterium buanense HM0024^{T} (FJ713810)
Chryseobacterium gleum NCTC 11432^{T} (X67852)
Chryseobacterium tenax EP105^{T} (AF493696)
Elizabethkingia meningoseptica IFO 12535^{T} (D14018)
Chryseobacterium koreense NBRC 103027^{T} (AB681907)
Candidatus Amoebinatus massiliensis CIP 107750T^{T} (AF531765)
Chryseobacterium antarcticum AT1013^{T} (AY553293)
Chryseobacterium taklimakanense X-65^{T} (EU718058)
Spodiobacter cordis PAGU 1467^{T} (AB818539)
Cloacibacterium normanense CCUG 46293^{T} (AJ575430)
Riemerella anatipestifer strain ATCC 11845^{T} (U60101)
Amniculibacterium aquaticum KYPW7^{T} (MG603666)
Bergeyella zoohelcum ATCC 43767^{T} (M93153)
Daejeonia ginsenosidivorans NP5^{T} (KT950745)
0.020

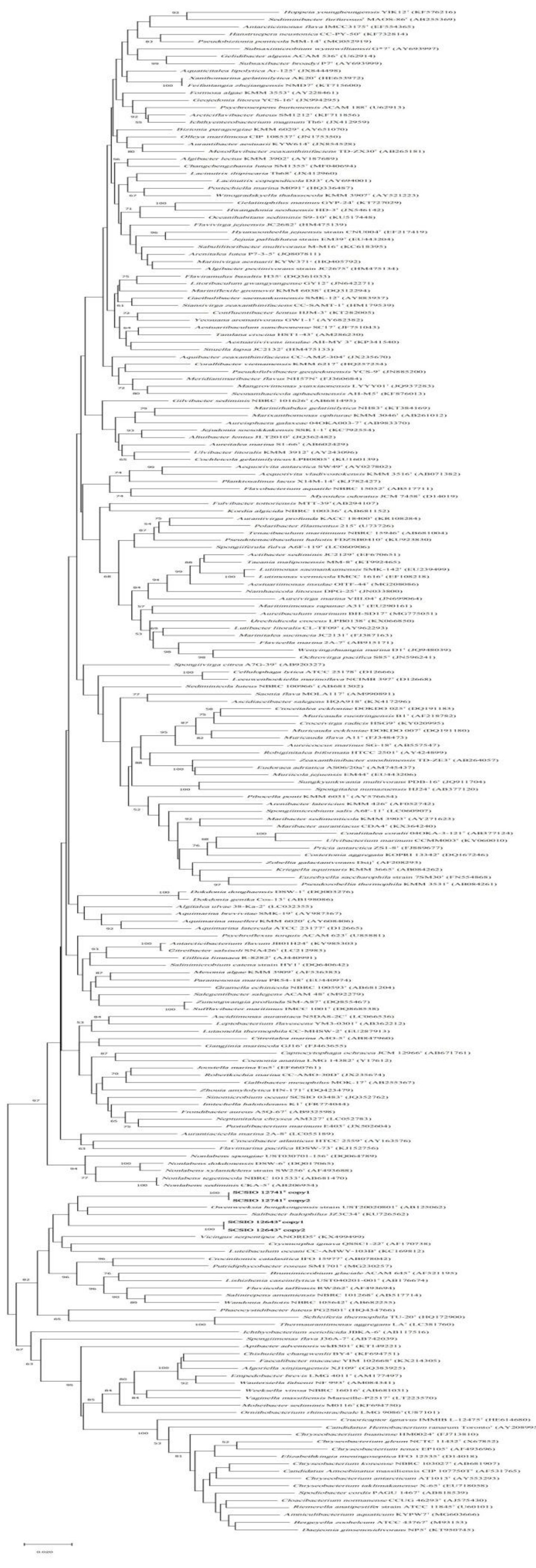

584

Figure 21. Neighbour-joining phylogenetic tree reconstructed based on 16S rRNA gene sequences, showing the position of strains SCSIO 12643^{T}, SCSIO 12741^{T} and other type species within families in the order Flavobacteriales. Two 16S rRNA gene copies of strain SCSIO 12643^{T} and SCSIO 12741^{T} were included in phylogenetic analysis. Only bootstrap values (percentages of 1000 replications) >50% were shown. The strain characterized in this study is shown in bold type. All ambiguous positions were removed for each sequence pair (pairwise deletion option). There were a total of 1661 positions in the final dataset. Bar, 0.02 changes per nucleotide position.

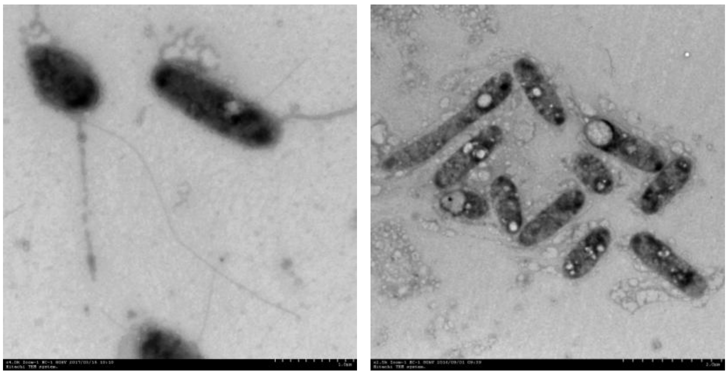

Figure 22. Transmission electron microscope photographs of strains SCSIO 12643^{T} (left), and SCSIO 12741^{T} (right).

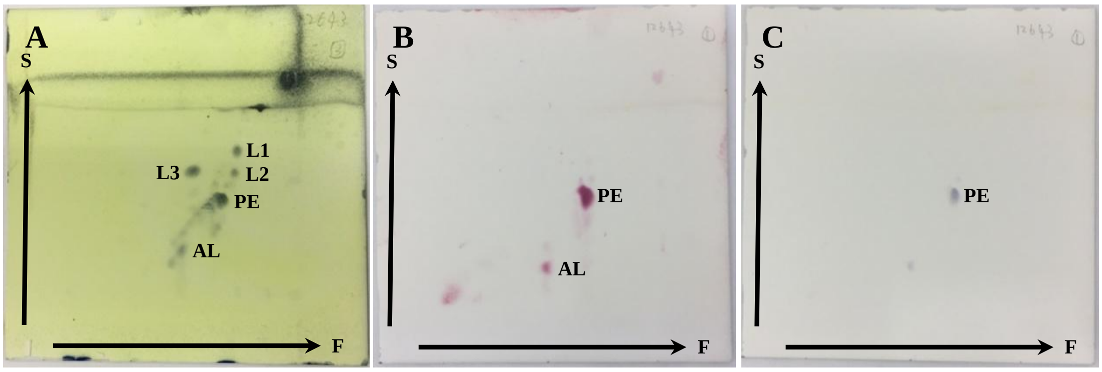

Figure 23. Two-dimensional thin layer chromatogram of polar lipids of strain SCSIO 12643^{T}. Chromatograms were run as follows: Silica Gel 60 thin-layer plates (10 by 10 cm) were spotted with 10 μL of a whole-cell lipid extract. Chloroform/methanol/water (65:25:4, by vol.) was used in the first direction, and chloroform/acetic acid/methanol/water (80:18:12:5, by vol.) was used in the second direction. A, The plate was sprayed with molybdatophosphoric acid; B, The plate was sprayed with ninhydrin reagent; C, The plate was sprayed with molybdenum blue reagent; PE, phosphatidylethanolamine; AL, unidentified aminolipid; L, unidentified polar lipid. F, first dimension of thin layer chromatogram; S, second dimension of thin layer chromatogram.

Description of *Pocilloporicola* gen. nov.

Pocilloporicola (Po.cil.lo.po.ri'co.la N.L. gen. n. *pocillopori*, pertaining to the coral *Pocillopora damicornis*; L. masc./fem. suff.–*cola*, inhabitant, dweller; N.L. masc. n. *Pocilloporicola*, coral *Pocillopora*-dweller).

Strain SCSIO 12741^T showed the highest 16S rRNA gene similarity (88.90%) to *Owenweeksia hongkongensis*. According to the 16S rRNA gene-based phylogeny, the strain is placed at a distinct clade from type species within families in the order Flavobacteriales, and clustered with *Owenweeksia hongkongensis* (Schleiferiaceae), *Salibacter halophilus* (Cryomorphaceae), and SCSIO 12643^T (Fig. 21). The POCP value between the genome of strain SCSIO 12741^T and *Owenweeksia hongkongensis*, *Salibacter halophilus*, and SCSIO 12643^T is 29.86%, 33.80%, and 36.68%, respectively. Thus, we propse it represents a novel genus.

Gram-stain-negative, aerobic, rod-shaped (Fig. 22). Negative for catalase and oxidase. Requiring seawater for growth. The major respiratory quinone is MK-6. The main cellular fatty acids are summed feature 3 ($C_{16:1}\omega 7c/C_{16:1}\omega 6c$), summed feature 4 (iso-$C_{17:1}$ I/anteiso-B), iso-$C_{15:0}$, and iso-$C_{17:0}$ 3-OH. The polar lipids present are diphosphatidylglycerol, phosphatidylethanolamine, and an unidentified aminolipid (Fig. 24). The G+C content of the genomic DNA of the type strain is 44.21%. The type species is *Pocilloporicola aurantiacus*.

Description of *Pocilloporicola aurantiacus* sp. nov.

Pocilloporicola aurantiacus (au.ran.ti.a'cus N.L. masc. adj. *aurantiacus*, orange-coloured).

In addition to the characteristics reported for the genus, colonies are circular, orange with entire margins, 0.5 mm in diameter on marine agar after cultivation for 4 days at 25 °C. Growth occurs at 20–35 °C (optimum, 30 °C), at pH 6–8 (optimum, pH 7) and with 3–3.7% (w/v) NaCl concentrations. Negative for hydrolysis of gelatin and starch, urease activity, and nitrate reduction. Positive for alkaline phosphatase, esterase (C4), esterase lipase (C8), leucine arylamidase, valine arylamidase, cystine arylamidase, trypsin, *α*-chymotrypsin, acid phosphatase, and naphthol-AS-BI-phosphohydrotase, but negative for lipase (C14), *α*-galactosidase, *β*-galactosidase, α-glucosidase, *β*-glucosidase, *α*-mannosidase, *β*-fucosidase, N-acetyl-*β*-glucosaminidase, and *β*-glucuronidase. Dextrin, D-maltose, D-trehalose, D-cellobiose, gentiobiose, sucrose, D-turanose, stachyose, D-raffinose, *α*-D-lactose, D-melibiose, *β*-methyl-D-glucoside, N-acetyl-*β*-D-mannosamine, N-acetyl-D-glucosamine, N-acetyl-D-galactosamine, *α*-D-glucose, D-mannose, D-galactose, 3-methyl glucose, D-fucose, L-fucose, L-rhamnose, inosine, D-sorbitol, D-mannitol, D-arabitol, myo-inositol, D-glucose-6-PO_4, D-fructose-6-PO_4, glycyl-L-prolin, pectin, D-galacturonic acid, L-galactonic acid lactone, glucuronamide, D-saccharic acid, acetoacetic acid, propionic acid, citric acid, L-malic acid, formic acid, and acetic acid are utilized as sole carbon and energy sources but D-salicin, N-acetyl neuraminic acid, D-fructose, L-arginine, L-aspartic acid, L-pyroglutamic acid, glycerol, D-aspartic acid, gelatin, L-serine, mucic acid, quinic acid, D-serine, L-alanine, methyl pyruvate, L-glutamic acid, L-histidine, *α*-keto-glutaric acid, D-gluconic acid, D-glucuronic acid, *α*-keto-butyric acid, D-malic acid, *p*-hydroxy-phenylacetic acid, D-lactic acid methyl ester,

α-hydroxy-butyric acid, L-lactic acid, bromo-succinic acid, tween 40, γ-amino-butryric acid, and β-hydroxy-D,L butyric acid are not. Inhibited by 1% sodium lactate, fusidic acid, troleandomycin, rifamycin SV, lincomycin, guanidine HCl, vancomycin, and lithium chloride but not by minocycline, niaproof 4, tetrazolium violet, tetrazolium blue, potassium tellurite, sodium bromate, aztreonam, nalidixic acid, and sodium butyrate. The type strain is SCSIO 12741^{T}.

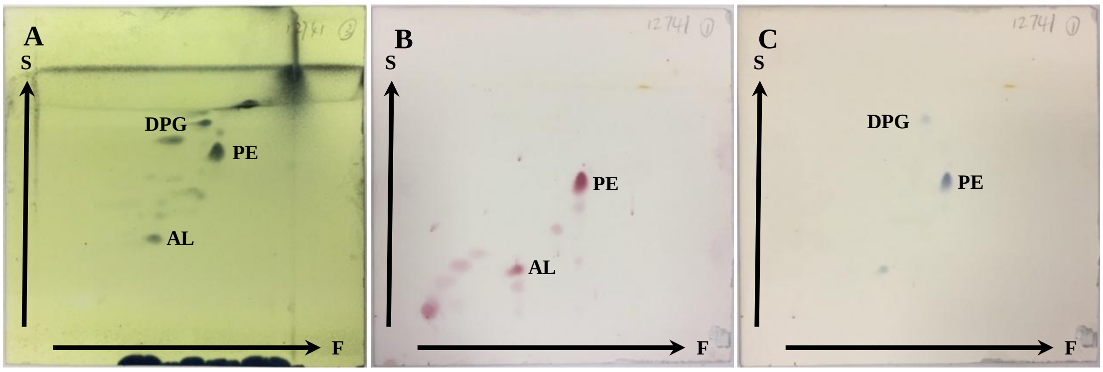

Figure 24. Two-dimensional thin layer chromatogram of polar lipids of strain SCSIO 12741^{T}. Chromatograms were run as follows: Silica Gel 60 thin-layer plates (10 by 10 cm) were spotted with 10 µL of a whole-cell lipid extract. Chloroform/methanol/water (65:25:4, by vol.) was used in the first direction, and chloroform/acetic acid/methanol/water (80:18:12:5, by vol.) was used in the second direction. A, The plate was sprayed with molybdatophosphoric acid; B, The plate was sprayed with ninhydrin reagent; C, The plate was sprayed with molybdenum blue reagent; DPG, diphosphatidylglycerol; PE, phosphatidylethanolamine; AL, unidentified aminolipid. F, first dimension of thin layer chromatogram; S, second dimension of thin layer chromatogram.
