# Supplementary material for: Cultured Bacteria Provide Insight into the Functional Potential of the Coral-Associated Microbiome
Source: mSystems. 2022 Jun 13;7(4):e00327-22. doi: 10.1128/msystems.00327-22 (PMC9426491; doi:10.1128/msystems.00327-22)
Supplement: TABLE S3 [file msystems.00327-22-st003.docx]

**TABLE S3** Partial hydrolase activities and carbon sources utilization of nine novel species, and the numbers of glycoside degrading enzymes detected in their genomes.

|  |  | **SCSIO 12603** | **SCSIO 12610** | **SCSIO 12643** | **SCSIO 12664** | **SCSIO 12696** | **SCSIO 12741** | **SCSIO 12827** | **SCSIO 12839** | **SCSIO 12844** |
| --- | --- | --- | --- | --- | --- | --- | --- | --- | --- | --- |
| API ZYM | α-galactosidase | - | - | - | + | - | - | + | - | - |
|  | β-galactosidase | - | - | - | + | - | - | + | - | - |
|  | β-glucuronidase | - | - | - | - | - | - | - | - | - |
|  | α-glucosidase | - | - | - | - | + | - | + | - | - |
|  | β-glucosidase | - | - | - | - | + | - | + | - | - |
|  | *N*-acetyl-β-glucosaminidase | - | - | + | + | - | - | - | - | - |
|  | α-mannosidase | - | - | - | - | - | - | + | - | - |
|  | β-fucosidase | - | - | - | - | - | - | - | - | - |
|  | Esterase | + | + | + | + | + | + | + | - | + |
|  | Esterase lipase | + | + | + | + | + | + | + | + | + |
|  | Lipase | + | - | - | + | + | - | + | - | - |
|  | Trypsin | + | + | + | + | + | + | + | + | - |
|  | α-chymotrypsin | + | + | - | + | + | + | + | - | - |
| oligo-saccharide | Dextrin | + | + | + | + | + | + | + | + | + |
|  | D-Maltose | + | + | - | + | + | + | + | - | - |
|  | D-Trehalose | - | + | + | + | + | + | - | + | + |
|  | D-Cellobiose | + | + | + | + | + | + | + | + | + |
|  | Gentiobiose | + | + | + | + | + | + | + | + | + |
|  | Sucrose | + | + | + | + | + | + | + | + | + |
|  | D-Turanose | + | - | + | - | + | + | + | + | + |
|  | Stachyose | + | + | + | + | + | + | + | + | + |
|  | D-Raffinose | + | + | + | + | + | + | + | + | + |
|  | α-D-Lactose | - | + | + | + | + | + | + | + | + |
|  | D-Melibiose | + | + | + | + | + | + | + | + | + |
|  | β-Methyl-D-Glucoside | - | + | + | + | + | + | - | - | + |
|  | D-Salicin | + | + | + | + | + | - | + | + | - |
| monosaccharide | N-Acetyl-D-Glucosamine | - | + | + | + | + | + | - | + | + |
|  | N-Acetyl-β-D-Mannosamine | - | + | - | + | + | + | - | + | + |
|  | N-Acetyl-D-Galactosamine | + | + | + | + | + | + | - | + | + |
|  | N-Acetyl Neuraminic Acid | - | - | - | - | - | - | - | - | - |
|  | α-D-Glucose | + | - | + | + | + | + | + | + | + |
|  | D-Mannose | + | + | + | + | + | + | + | + | - |
|  | D-Fructose | + | - | + | + | + | - | + | + | + |
|  | D-Galactose | + | + | + | + | + | + | + | + | - |
|  | 3-Methyl Glucose | - | + | + | + | + | + | + | + | - |
|  | D-Fucose | + | + | + | + | + | + | + | + | + |
|  | L-Fucose | + | + | + | + | + | + | + | + | - |
|  | L-Rhamnose | + | + | + | + | + | + | + | + | + |
|  | D-Glucose-6-PO4 | - | - | - | - | + | + | - | + | + |
|  | D-Fructose-6-PO4 | - | + | - | + | + | + | + | + | - |
|  | D-Galacturonic Acid | - | + | - | + | - | + | - | + | - |
|  | L-Galactonic Acid Lactone | - | + | + | - | + | + | - | + | - |
|  | D-Gluconic Acid | - | - | - | - | - | - | - | - | - |
|  | D-Glucuronic Acid | + | - | - | - | + | - | - | - | - |
|  | Glucuronamide | + | + | + | - | + | + | + | + | + |
|  | Mucic Acid | - | - | - | - | - | - | + | - | - |
|  | Quinic Acid | + | - | - | - | + | - | + | - | - |
|  | D-Saccharic Acid | + | + | + | + | + | + | + | + | + |
| polysaccharide | starch | + | + | - | - | + | - | + | - | - |
|  | pectine | - | + | + | + | + | + | - | + | + |
| glycoside degrading enzymes (GDEs) | | 14 | 15 | 11 | 10 | 14 | 12 | 12 | 18 | 9 |
