# Supplementary material for: Cultured Bacteria Provide Insight into the Functional Potential of the Coral-Associated Microbiome
Source: mSystems. 2022 Jun 13;7(4):e00327-22. doi: 10.1128/msystems.00327-22 (PMC9426491; doi:10.1128/msystems.00327-22)
Supplement: TABLE S4 [file msystems.00327-22-st004.docx]

**TABLE S4** Genome capabilities of *Pocillopora damicornis* and bacterial symbionts in the biosynthesis of amino acids and vitamins.

| **Nutrients** | ***Pocillopora damicornis*** | **coral-associated bacteria** |
| --- | --- | --- |
| **Vitamin** |  |  |
| Biotin | - | + |
| Cobalamin | - | + |
| Folate | - | + |
| Pantothenate | - | + |
| Riboflavin | - | + |
| Thiamine | - | + |
| Pyridoxine | - | + |
| **Amino acid** |  |  |
| Alanine | + | + |
| Arginine | + | + |
| Asparagine | + | + |
| Aspartate | + | + |
| Cysteine | + | + |
| Glutamine | + | + |
| Glutamate | + | + |
| Glycine | + | + |
| Histidine | + | + |
| Isoleucine | - | + |
| Leucine | - | + |
| Lysine | - | + |
| Methionine | - | + |
| Phenylalanine | - | + |
| Proline | + | + |
| Serine | + | + |
| Threonine | + | + |
| Tryptophan | - | + |
| Tyrosine | - | + |
| Valine | - | + |
