# Supplementary material for: Cultured Bacteria Provide Insight into the Functional Potential of the Coral-Associated Microbiome
Source: mSystems. 2022 Jun 13;7(4):e00327-22. doi: 10.1128/msystems.00327-22 (PMC9426491; doi:10.1128/msystems.00327-22)
Supplement: TABLE S5 [file msystems.00327-22-st005.docx]

**TABLE S5** Yields of vitamins in extracellular and intracellular of strains SCSIO 12696 and SCSIO 12664.

| Strain No. | Folic acid (ng mL^-1^) | Biotin (ng mL^-1^) | Cyanocobalamin (ng mL^-1^) | Thiamine (ng mL^-1^) | Riboflavin (ng mL^-1^) | Pantothenic acid (ng mL^-1^) | Pyridoxine |
| --- | --- | --- | --- | --- | --- | --- | --- |
| SCSIO 12696 (extracellular) | 2.37 ± 1.25 | 3.00 ± 0.89 | - | 21.09 ± 18.98 | 60.74 ± 36.87 | 255.00 ± 121.91 | - |
| SCSIO 12696 (intracellular) | 18.68 ± 7.08 | 0.20 ± 0.07 | 0.11 ± 0.05 | nd | nd | nd | nd |
| SCSIO 12664 (extracellular) | 96.57 ± 37.08 | 0.89 ± 0.31 | - | - | 118.59 ± 35.45 | - | - |
| SCSIO 12664 (intracellular) | nd | nd | nd | nd | nd | nd | nd |
